# Colorectal cancer pathogenesis, oncogenic signaling networks and targeted therapeutic advances

**DOI:** 10.1186/s43556-026-00433-4

**Published:** 2026-03-14

**Authors:** Yue Chen, Jiaqi Zhang, Yi Ding, Fang Zhu, Yinnan Chen

**Affiliations:** 1 Center for Gut Microbiome Research, Med-X Institute, The First Affiliated Hospital of Xi’an Jiaotong University, Xi’an, Shaanxi China; 2 Department of High Talent, The First Affiliated Hospital of Xi’an Jiaotong University, Xi’an, Shaanxi China; 3 Hubei Province Key Laboratory of Precision Radiation Oncology, Wuhan, China; 4 Cancer Center, Union Hospital, Tongji Medical College, Huazhong University of Science and Technology, Wuhan, China

**Keywords:** Colorectal Cancer, Targeted Therapy, Pathogenesis, Targeted inhibitors

## Abstract

Colorectal cancer (CRC) constitutes a prominent global health burden, being the third most frequently diagnosed malignancy in terms of incidence and the second leading cause of cancer-associated death across the globe. Malignant transformation of colonic epithelial cells stems from the intricate dysregulation of intracellular signal transduction networks. Although targeted therapies have substantially improved patient survival relative to traditional treatments, the complexity of the molecular networks driving carcinogenesis continues to limit the overall prognosis. This review delineates the core signaling cascades governing CRC initiation and progression, with emphasis on the molecular hallmarks of the disease. Drawing on a growing body of high-quality preclinical and clinical evidence, we summarize currently available targeted agents and critically evaluate their underlying mechanisms of action and clinical curative effects, and inherent limitations within the contemporary therapeutic landscape. In addition, we discuss how recent advances in immune checkpoint inhibitors (ICIs) along with a deeper understanding of the tumor microenvironment are shaping global clinical guidelines and revealing promising new targets and combinatorial strategies. In summary, expanding insights into oncogenic signaling pathways are guiding the development of novel treatments and enabling the identification of key elements amenable to pharmacological intervention. Ultimately, this review aims to support the rational design of precise and personalized therapeutic strategies to improve CRC prognosis.

## Introduction

Colorectal cancer (CRC) presents a substantial and increasing burden on global health, representing the third most prevalent malignancy and the second highest cause of death from cancer globally [1]. From 1990 to 2019, the global annual incidence of CRC saw a dramatic rise, surging from 0.84 million to 2.17 million cases-an increase of over 150%, while related deaths increased from 0.52 million to 1.09 million [2]. Based on 2022 statistics released, the global incidence of CRC reached an estimated 1.93 million new cases, with the disease causing more than 900,000 deaths worldwide, according to 2022 WHO and Chinese National Cancer Center data, China recorded 517,100 new CRC cases (26.8% of global total) and 240,000 deaths (26.5%) [1, 3]. As the nation’s second most common and fourth deadliest cancer, it poses major prevention challenges. According to the “Cancer Statistics, 2026” report, CRC in the United States is projected to have 158,850 new cases and 55,230 deaths in 2026 [4]. This risk is compounded by established factors, including lifestyle habits, alcohol consumption, and gut microbiota dysbiosis [5–7]. Projections estimate that by 2030, new global CRC cases will exceed 2.2 million, with the overall disease burden expected to increase by 60% [8].

CRC develops through a complex interplay of genetic and environmental factors, converging on key tumor growth and progression pathways. Critical molecular drivers include mutations in adenomatous polyposis coli (APC), KRAS, BRAF, and TP53, which aberrantly activate the Wnt, epidermal growth factor receptor (EGFR), and PI3K/AKT signaling cascades. Tumorigenesis is further promoted by alterations associated with chromosomal instability (CIN), microsatellite instability (MSI), and the CpG island methylator phenotype (CIMP) [9]. The complexity and interdependence of these mechanisms profoundly influence CRC initiation and progression as well as drug treatment efficacy.

Considering the substantial burden of CRC, the development and implementation of effective therapeutics is an urgent priority. The past two decades have witnessed significant research into the molecular pathogenesis of CRC, which has accelerated dramatically [10, 11]. This progress has directly ushered in the era of targeted therapy, beginning with the Food and Drug Administration (FDA) approval of cetuximab and bevacizumab in 2004. Since then, a succession of targeted drugs have been approved, with many more currently under investigation [12]. Together with the ongoing advances in medical science, these developments have driven the emergence of more precise and personalized therapeutic approaches, leading to significant improvements in patient survival [13]. Contemporary management strategies range from conventional treatments like surgery, chemotherapy, and radiotherapy to more advanced modalities such as targeted therapy, immunotherapy, and intestinal flora modulation. This study examined the molecular evolution of CRC treatment, focusing on the mechanisms, impact, and future direction of targeted therapeutics.

## Molecular pathogenesis: foundation for targeted therapy

### Origin and evolution of colorectal cancer

**Fig. 1:**
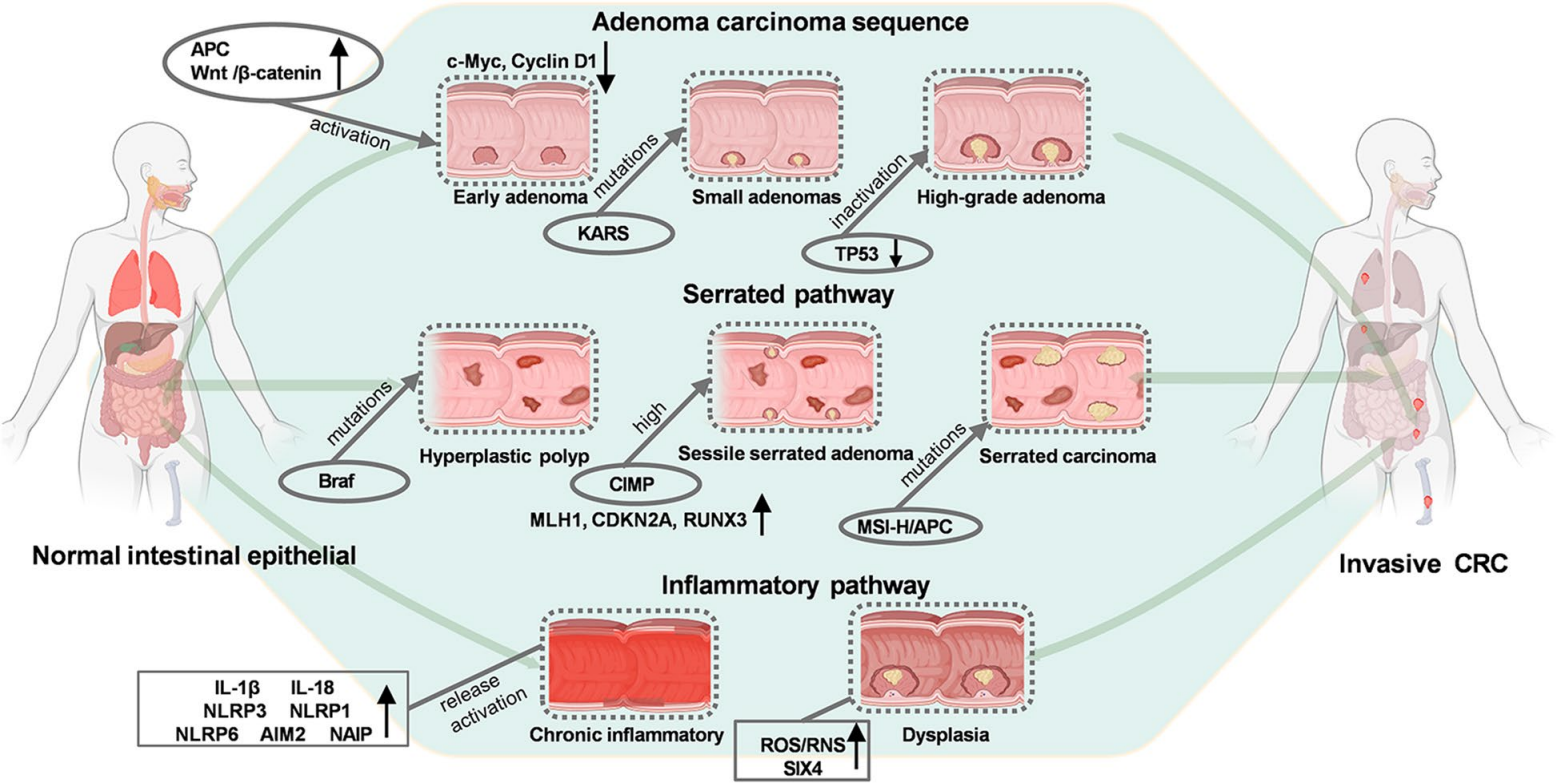
The pathological process of the occurrence and development of CRC. Diagram depicting the three main pathways in CRC pathogenesis the adenoma—carcinoma sequence, serrated pathway, and inflammatory pathway. The adenoma—carcinoma sequence starts with normal intestinal epithelium, progressing to small adenomas via APC/Wnt/β—catenin activation, then to early, intermediate, and high—grade adenomas through mutations/loss of KRAS, and TP53, respectively. The serrated pathway begins with hyperplastic polyps, advancing to sessile serrated adenomas (via BRAF mutant) and then to serrated carcinomas (with high CIMP). The inflammatory pathway involves chronic inflammation leading to dysplasia, with ROS/RNS and TP53 mutations contributing, ultimately resulting in invasive CRC in all pathways

Developing effective targeted therapies for CRC necessitates a deeper understanding of its origin and progression. CRC is a progressive and heterogeneous disease with a multistep development through a series of cellular and molecular changes, progressing from normal intestinal epithelial cells through preneoplastic lesions to invasive carcinoma and ultimately metastatic disease. This progression is driven by the sequential accumulation of genetic mutations, epigenetic modifications, and dysregulated signaling pathways, which collectively dismantle cellular homeostasis, proliferation, and apoptosis [14–16]. Clinically, CRC pathogenesis follows three distinct evolutionary pathways: the conventional adenoma-carcinoma sequence, the serrated pathway, and the inflammation-driven pathway. Each pathway is characterized by unique initiating events, specific molecular alterations, and pathological progression patterns [17–19] (Fig. 1). External factors such as pathogenic bacteria can also contribute to the occurrence of CRC.

#### Adenoma-carcinoma sequence

The adenoma-carcinoma sequence, which accounts for approximately 65–70% of sporadic CRCs, follows a well-defined progression: normal epithelium—early adenoma—small adenoma (low-grade dysplasia)—large adenoma (high-grade dysplasia)—invasive adenocarcinoma [13]. This sequence is fundamentally driven by CIN, resulting in structural and numerical chromosomal abnormalities that promote the accumulation of driver gene alterations [20]. Loss of APC destabilizes the β-catenin destruction complex, resulting in β-catenin stabilization and accumulation within the cytoplasm and nucleus [21]. This subsequently activates target genes (e.g., c-Myc, Cyclin D1), promoting uncontrolled cell proliferation and the formation of early adenomas [22]. Concomitantly, activating mutations in the oncogene KRAS, present in approximately 40% of CRCs, promote adenoma growth by constitutively activating the MAPK/ERK pathway, whereas inactivation of the tumor suppressor gene TP53 facilitates the progression of adenomas to CRCs. The CIN-positive subtype of CRC primarily develops through the adenoma-carcinoma pathway [23]. This sequence is the primary driver of CIN-positive CRC, a subtype associated with aggressive clinical behavior and a high incidence of distant metastasis [13, 23].

Knowledge of these molecular alterations has directly translated into successful clinical therapies. EGFR-targeting monoclonal antibodies, such as cetuximab and panitumumab, are the standard of care for metastatic KRAS wild-type CRC by inhibiting the MAPK pathway [24]. Notably, KRAS mutations that confer resistance to these anti-EGFR agents can now be directly targeted: KRAS G12C-specific inhibitors (e.g., sotorasib and adagrasib) have recently been approved for patients with this precise mutation [25]. Furthermore, the initiating Wnt/β-catenin pathway is under active investigation. Preclinical agents like β-catenin/TCF interaction inhibitors (e.g., CWP232291) and Porcupine inhibitors (e.g., WNT974) demonstrate promising efficacy and are currently advancing through clinical trials for BRAF-mutant metastatic CRC (mCRC) (NCT02278133) [26–28].

#### Serrated pathway

In addition to the adenoma- carcinoma sequence, the serrated pathway, which accounts for 15%- 20% of CRCs, is a major alternative molecular mechanism, primarily driven by epigenetic abnormalities. This epigenetically driven progression evolves from serrated polyps: normal epithelium-hyperplastic polyp-sessile serrated adenoma (SSA, the key precursor)-serrated adenocarcinoma [29]. Unlike the conventional sequence, the serrated pathway is distinctively characterized by two hallmark early molecular events: oncogenic BRAF mutations and CIMP [30]. Research also find S100A14 as a potential biomarker of the colorectal serrated pathway [31].

The activation of the BRAF V600E mutation, which is present in approximately 80% of serrated lesions, is the key initiating event in the serrated pathway. This mutation constitutively stimulates the MAPK/ERK signaling axis, mediating survival and growth signals that ensure proliferation and inhibit programmed cell death [32]. Concurrently, CIMP, which is characterized by the widespread hypermethylation of CpG islands in tumor suppressor gene promoters (e.g., MLH1, CDKN2A, and RUNX3), induces transcriptional silencing, disrupting cell cycle control and DNA mismatch repair [33]. This dual molecular alteration (BRAF mutation + CIMP) drives the progression from hyperplastic polyps to SSAs and invasive carcinoma [34]. CIMP is closely linked to this pathway: approximately 75% of SSAs and 90% of serrated adenocarcinomas exhibit CIMP-High (CIMP-H) status [35]. This distinction, along with the rare incidence of APC mutations and the frequent occurrence of microsatellite instability-High (MSI-H) status, often resulting from methylation-mediated MLH1 silencing, defines the unique biology of serrated CRCs [36].

#### Inflammation-driven pathway

The third key mechanism, the inflammation-driven pathway, accounts for 5%−10% of CRCs, and is strongly associated with chronic intestinal inflammation, particularly in individuals diagnosed with inflammatory bowel disease (IBD), which comprises ulcerative colitis and Crohn's disease [37]. Prolonged inflammation disrupts mucosal homeostasis through repeated epithelial damage and repair cycles. This leads to a procarcinogenic microenvironment, defined by three key features: elevated reactive oxygen species, increased reactive nitrogen species, and a milieu of proinflammatory cytokines [38]. These mediators induce severe oxidative stress and DNA damage, accelerating the progressive buildup of genetic and epigenetic modifications. The overall process promotes the progression from chronic inflammation to dysplasia and, ultimately, to colitis-associated CRC (CAC) [39].

Inflammasomes are key cytoplasmic immune complexes that respond to cellular stress, mediating inflammation and cell death. In various colitis-associated CRCs, inflammasome activation and the release of cytokines like interleukin (IL)−1β and IL-18 are observed [40]. Sensors such as NLRP3, NLRP1, NLRP6, and Pyrin are activated to exert a protective role against this specific subtype of CRC [41]. Independently, other inflammasome sensors (e.g., AIM2, NLRC4, NAIP) also function. A key oncogenic mechanism involves the transcription factor SIX4, which is upregulated during the colitis-adenoma-carcinoma sequence [42]. SIX4 is activated by IL-6/STAT3 signaling and subsequently interacts with c-Jun to establish a positive feedback loop that amplifies IL-6 transcription and sustains chronic inflammation. Additionally, SIX4 promotes CRC progression by inducing ΔNp63 expression and activating cancer stemness pathways. Therapeutically, targeting SIX4 mitigates colitis and adenoma formation [43]. Consistent with the molecular data, a population study (NHANES 2001–2020) found that inflammatory indices, specifically, an elevated neutrophil-to-lymphocyte ratio (NLR) combined with a low lymphocyte-to-monocyte ratio (LMR), are linked to an increased risk of CRC. Furthermore, a low LMR, a high neutrophil-to-platelet ratio (NPR), and a high systemic immune-inflammation index have been associated with worse clinical outcomes, underscoring their potential as noninvasive prognostic biomarkers [44].

#### Tumor microenvironment (TME) with CRC

TME constitutes an intricate ecosystem, where the heterogeneous cellular composition engages in active interplay with acellular elements, critically modulating cancer progression and anti-tumor immunity. Cellular and non-cellular components within the TME collectively shape disease progression. These include malignant cells, infiltrating immune cells, soluble factors (e.g., cytokines), and the extracellular matrix (ECM). Immune cells within the CRC microenvironment- including tumor-associated macrophages (TAMs), myeloid-derived suppressor cells (MDSCs), tumor-infiltrating T cells, tumor-associated neutrophils, regulatory T cells (Tregs), and fibroblasts- are critically involved in disease pathogenesis. Cytokines, such as interleukin-1 (IL-1), IL-11, IL-22, and tumor necrosis factor (TNF), which are produced by myeloid cells, T cells, and fibroblasts, contribute to CRC progression by activating signaling pathways such as nuclear factor kappa B (NF-κB) [45, 46]. Studies have found that the expression of Dipeptidyl peptidase VII (DPP7, also designated DPP2) in TAMs is elevated in CRC. High expression of this gene is associated with metastasis and lower survival rates in multiple clinical cohorts. Further research has revealed that DPP7 promotes M2 polarization of TAMs by enhancing fatty acid oxidation and increasing adenosine triphosphate (ATP) production, thereby exacerbating CD8⁺ T cell exhaustion and generating immunosuppression [47]. For MDSCs, they promote the expression of chemokines through Gasdermin C (GSDMC)-mediated pyroptosis of tumor cells, further recruiting MDSCs to the tumor microenvironment and thus promoting CRC progression [48]. Further research demonstrated that in CRC, the microbial metabolite 4-hydroxyphenylacetic acid (4-HPA) contributes to tumor progression by activating the JAK2/STAT3 signaling pathway. This activation leads to the transcriptional upregulation of CXCL3, which in turn facilitates the recruitment and infiltration of polymorphonuclear myeloid-derived suppressor cells (PMN-MDSCs) into the tumor microenvironment. The accumulated PMN-MDSCs subsequently suppress the antitumor activity of CD8⁺ T cells, thereby promoting CRC progression in vivo [49]. Tregs are recruited to the tumor microenvironment by cytokines (CCL17 and CCL20) to exert immunosuppressive effects. However, recent studies have shown that SIRT1 promotes the upregulation of CX3CL1 in CRC cells, which enhances Treg function and suppresses antitumor immunity [50]. Research has also found that the phenotypic and functional homeostasis of Treg cells is maintained by lactate through Foxp3-dependent regulation of RNA splicing [51]. Elevated lactate concentrations inhibit the activity of nuclear factor of activated T cells (NFAT) in T cells and natural killer (NK) cells, weaken interferon-γ (IFN-γ) production, and thus promote immune evasion [52].

In a phase II, open-label, single-arm cohort trial evaluating regorafenib combined with (ICIs (specifically sintilimab) as salvage treatment for MSS mCRC, the regimen demonstrated a median overall survival (OS) of 14.1 months, an objective response rate (ORR) of 21.4%, and a disease control rate (DCR) of 63.1%. Patients with wild-type RAS/RAF tumors derived greater clinical benefit, achieving a median OS of 23.3 months. The combination therapy was well-tolerated with manageable safety. The study suggests that this regimen provides a promising later-line treatment option for MSS mCRC patients [53]. A separate investigation examined the therapeutic outcomes and safety profiles of sequential versus concurrent delivery of ICIs and radiotherapy in patients with solid tumors. Results indicated that administering ICIs after radiotherapy significantly enhanced OS (HR = 0.81) and progression free survival (PFS) (HR = 0.73). In contrast, the concurrent treatment approach did not demonstrate a statistically significant survival advantage. The sequential approach significantly improved PFS compared to the concurrent approach (HR = 0.81). There were no difference in grade ≥ 3 adverse events between the two groups [54].

#### Microbiota with CRC

Microbiota serve as significant external drivers of CRC, and promote tumorigenesis through multiple ways including immunosuppression, DNA damage, and activation of oncogene signaling pathways. The conditional pathogen *Alcaligenes faecalis* translocates to colon tissue during colitis, leading to a reduction in Peyer’s patch IgA⁺ B cells and immunosuppression. It also promotes carcinogenesis by increasing epithelial tight junction protein acetylation, disrupting their binding to β-catenin [55]. The genotoxin colibactin, produced by pks⁺ *Escherichia coli*, can directly cause host DNA double-strand breaks, and its mutational signature is commonly found in adjacent normal colon tissues [56, 57]. Studies have shown that a low-carbohydrate diet rich in soluble fiber can inhibit the colonization of pks⁺ *E. coli* and colibactin-mediated DNA damage by reducing intestinal inflammation, enhancing PPAR-γ signaling, and lowering nitrate levels, thereby preventing colon polyp formation [58]. *Fusobacterium nucleatum* is another opportunistic pathogen that promotes carcinogenesis of CRC. It forms biofilms via adhesins (such as FadA), disrupting intestinal microbiota homeostasis and creating a proinflammatory microenvironment. Mechanistically, it inhibits the cGAS-IFNβ pathway to impair CD8⁺ T cell immune responses, leading to immunotherapy resistance [59]. Simultaneously, the infection reprograms crypt cells, activating LY6A⁺ revival stem cells and promoting their transformation into cancer stem cells, accelerating carcinogenesis [60]. This bacterium promotes cancer through multiple synergistic pathways, including virulence factors, immune evasion, stem cell reprogramming, and metabolic interference [61]. *Fusobacterium mortiferum* produces 5-aminovaleric acid, a metabolite that promotes tumor development by inhibiting KDM6B, downregulating DKK2, and thereby activating the Wnt/β-catenin pathway [62]. *Solobacterium moorei* drives CRC progression by engaging integrin α2/β1 on host cells with its Cna B-type domain-containing cell wall protein. This engagement activates the oncogenic FAK-PI3K-AKT-mTOR-C-myc axis. Targeting this interaction by blocking integrin α2/β1 offers a potential strategy to mitigate its cancer-promoting effects [63].

Current treatment methods for similar microbial dysbiosis, pathogenic bacteria, or opportunistic pathogens include various approaches such as probiotics, prebiotics, and antibiotics to regulate the gut microbiota [64, 65]. However, for targeting specific pathogenic bacteria, treatments include targeted phage therapy and specific vaccines. For example, against intratumoral *Fusobacterium nucleatum*, researchers have used cationic polymers to selectively encapsulate phage heads, which can inhibit intracellular pathogenic bacteria while remaining intact [66]. Additionally, researchers have prepared a biomimetic nanodrug by fusing *Fusobacterium nucleatum* membranes with drug-loaded liposomes, which can selectively kill the bacteria within tumors without harming the intestinal flora [67].

Having detailed the distinct evolutionary pathways and microenvironmental influences that drive colorectal carcinogenesis, it becomes evident that a systematic molecular classification is essential to translate this biological complexity into clinical practice. The heterogeneity inherent in CRC’s origins necessitates precise molecular profiling to stratify patients, predict behavior, and select targeted interventions, thereby bridging the gap between mechanistic understanding and therapeutic application.

### Molecular profiling and classification of CRC

A variety of molecular classification systems for CRC have been established based on key molecular changes. The most widely adopted clinical framework classifies CRC based on CIN status, CIMP, and MSI. These molecular categories encompass the major genetic and epigenetic aberrations driving CRC and are essential for guiding precision treatment strategies [68].

#### Chromosomal Instability (CIN)

CIN is the most prevalent form of genomic instability in CRC and adenomas and is observed in 80%−85% of cases. It is characterized by extensive chromosomal copy number variations and structural abnormalities, resulting in the inactivation of tumor suppressor genes (APC and TP53) and oncogene activation (KRAS) [69]. KRAS encodes GTP-binding proteins capable of promoting cell survival, while APC loss of function disrupts β-catenin signaling, ultimately leading to adenoma formation [70]. CIN-positive CRCs are typically left-sided and microsatellite-stable (MSS), features associated with poor prognosis [71]. Notably, reduced expression of the F-box protein EMI1 has been shown to markedly induce CIN in colonic epithelial cells. This is accompanied by increased DNA damage and enhanced transformative capacity, highlighting the critical role of EMI1 in early CRC development and establishing it as a prospective biomarker enabling early-stage detection of and targeted intervention [72].

#### CpG Island Methylator Phenotype (CIMP)

CIMP is an epigenetic phenomenon characterized by widespread hypermethylation across multiple CpG sites. CIMP is stratified into high (CIMP-H; 15%−20% of CRCs) and low (CIMP-L) subtypes. CIMP-H is strongly associated with BRAF V600E mutations, proximal tumor location, and younger patient age, although classification criteria remain inconsistent [73]. CIMP-H drives CRC pathogenesis by silencing tumor suppressor genes; for example, MLH1 promoter methylation causes mismatch repair deficiency (dMMR) and subsequent MSI [74]. Clinically, CIMP-H CRCs show improved early-stage outcomes but poor metastatic survival and resistance to 5-fluorouracil (5-FU) chemotherapy [75]. Using cellular and murine models, a pivotal recent study has demonstrated that IDH mutations can directly induce the CIMP phenotype in CRC [76]. These IDH mutations cosegregate with BRAF mutations but are mutually exclusive with KRAS mutations or MSI. This breakthrough identifies the first genetic driver of this epigenetic mechanism in a common malignancy, refutes the aging-related epiphenomenon theory, and immediately highlights IDH-mutant tumors as a novel therapeutic target [76].

#### Microsatellite Instability (MSI)

MSI, detected in 12–15% of CRCs, results from dMMR, which impairs the repair of short repetitive DNA sequence [77]. Approximately 90% of MSI is sporadic (due to MLH1 methylation), and 10% is hereditary (Lynch syndrome) [78]. MSI-H tumors are typically proximal, poorly differentiated, and often harbor BRAF mutations [79]. Prognostically, MSI-H tumors are associated with improved survival in early-stage disease but exhibit poorer outcomes following chemotherapy in metastatic settings [80]. dMMR tumors exhibit a high tumor mutational burden (TMB), rendering them highly responsive to ICIs. While these tumors show a better prognosis in early stages, they exhibit worse outcomes in metastatic disease and potential resistance to 5-FU. A recent pivotal study reveals that *Clostridium butyricum* improves the effectiveness of anti-PD-1 therapy in both MSI-H and MSS tumors. This enhancement is achieved by modulating the GRP78-PI3K-AKT-NF-κB signaling pathway, which subsequently attenuates IL-6-mediated immunosuppressive microenvironment [81]. Remarkably, these phenotypes interact, as CIMP can induce MSI by silencing MMR genes through methylation [82]. Furthermore, MSI status not only informs immunotherapy but it has also been found that Werner syndrome RecQ helicase (WRN) can serve as a therapeutic target for MSI subtype of cancers. Inhibiting the helicase domain at specific WRN residues exhibits synthetic lethality in cancers with MSI-H [83]. Moreover, drugs targeting WRN are under active development [84, 85], with inhibitors such as HRO761 (NCT05838768) and VVD‑133,214 currently in clinical investigation [86].

Additionally, the Consensus Molecular Subtypes (CMS) represent the most influential molecular classification system for CRC. Based on gene transcriptomic features, it categorizes CRC into four distinct subtypes with significant biological and clinical heterogeneity. The CMS1 subtype (MSI-Immune) is characterized by MSI-H and high immune infiltration, showing sensitivity to immunotherapy. The CMS2 subtype (Canonical) is marked by activation of the WNT/MYC pathway and benefits from oxaliplatin-based chemotherapy and anti-EGFR therapy. The CMS3 subtype (Metabolic) frequently exhibits KRAS mutations and metabolic reprogramming. The CMS4 subtype (Mesenchymal) demonstrates epithelial-mesenchymal transition (EMT) and a pro-fibrotic tumor microenvironment, correlating with the poorest prognosis. This classification also provides an essential framework for guiding treatment decisions [87].

These molecular profiles provide a crucial framework for categorizing CRC, yet they ultimately exert their biological influence by dysregulating core cellular signaling pathways. Understanding these pathways is therefore essential to decipher how molecular alterations drive tumorigenesis and to identify actionable therapeutic targets. The following section details the key signaling cascades frequently disrupted in CRC.

### Signaling pathways in colorectal cancer tumorigenesis

**Fig. 2:**
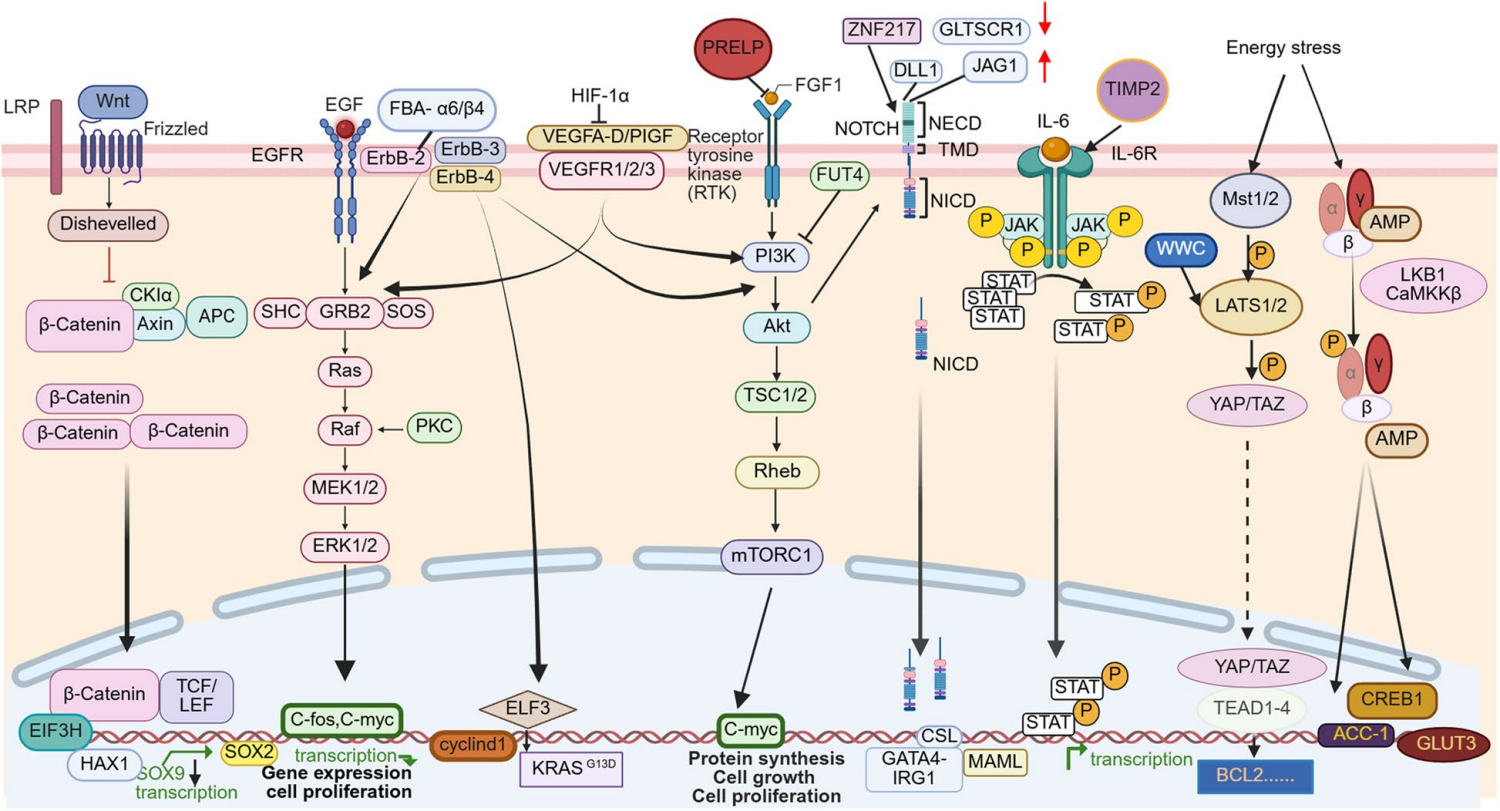
Signaling pathways involved in cellular regulation and proliferation. Schematic illustration of multiple signaling pathways that regulate gene expression, protein synthesis, cell growth, and proliferation. Pathways shown include Wnt/β—catenin, receptor tyrosine kinase (RTK)—mediated (e.g., ErbB family, PI3K-Akt-mTORC1), IL-6 -JAK -STAT, NOTCH, Hippo (Mst1/2-LATS1/2-YAP/TAZ), energy stress—related (AMP-activated), and VEGF-RAS-RAF-MEK-ERK pathways. These pathways converge on transcriptional regulation and cellular functions such as proliferation, growth, and metabolism, with key molecules highlighted at each step of signal transduction

Differences among molecular subtypes stem from the abnormal activation of various signaling pathways. The occurrence and development of CRC are jointly mediated by a complex, intertwined network encompassing the vascular endothelial growth factor (VEGF), Hippo, AMP-activated protein kinase (AMPK), Wnt/β-catenin, PI3K/AKT/mTOR, TGF-β, JAK/STAT, ErbB, and Notch pathways. The intricate crosstalk between these cascades drives the malignancy to varying degrees [88] (Fig. 2).

#### Wnt/β-catenin signaling pathway

The Wnt/β-catenin pathway fundamentally influences CRC initiation and progression by regulating critical processes, including cell proliferation, stemness, apoptosis, autophagy, metabolism, immunity, microenvironment modulation, drug resistance, and metastasis [88]. In CRC, Wnt ligand binding to the LRP5/6 and Frizzled (FZD) receptor complex recruits Dishevelled (Dvl), which inactivates the destruction complex (GSK3β, CKI, Axin, and APC) that normally targets β-catenin for degradation [89, 90]. Activation of Wnt signaling leads to β-catenin accumulation, nuclear import, and subsequent association with TCF/LEF1 factors, thereby inducing the transcription of oncogenic Wnt target genes. Conversely, when Wnt signaling is off, the destruction complex remains functional, resulting in low steady-state levels of β-catenin [91].

Previous studies have extensively summarized the role of the Wnt/β-catenin pathway in CRC [90, 92]. In recent years, advances in research and development have revealed novel regulatory mechanisms. For instance, the eukaryotic initiation factor 3 subunit H (EIF3H), which is overexpressed in CRC and essential for translational initiation, is transcriptionally induced by Wnt/β-catenin signaling [93]. EIF3H functions as a deubiquitinase for HAX1, stabilizing it against βTrCP-mediated degradation. This stabilization enhances the RAF1-MEK1-ERK1 complex, promoting ERK1/2 phosphorylation and subsequent oncogenic signaling [94]. In addition to the EIF3H -mediated regulatory branch, the Wnt/β-catenin target gene SOX9 exhibits a context-specific role in CRC. Simultaneous inactivation of APC and SOX9 drives tumors toward a highly invasive phenotype, accompanied by EMT and increased SOX2 expression [95]. Loss of SOX9 accelerates metastasis and predicts poor prognosis for CRC [95]. However, in gastric cancer, SOX9 promotes proliferation and stemness, with its regulatory effects mediated through the Wnt signaling pathway [96].

In addition, the Wnt/β-catenin pathway can also form a cross-regulatory network with the VEGF and EGFR pathways through clear molecular mechanisms. The activation of the Wnt pathway can indirectly promote the synthesis and secretion of VEGF by up-regulating the transcriptional expression of downstream target genes, thereby regulating the process of tumor angiogenesis. The Wnt pathway can form a synergistic effect with the ERK signal downstream of the EGFR pathway to regulate the expression of proliferation-related genes such as Cyclin D1, and synergistically promote the malignant progression of tumors.

#### VEGF signaling pathway

The VEGF pathway is crucial for CRC angiogenesis and metastasis. The VEGF ligands (VEGFA-D and PlGF) signal through three tyrosine kinase receptors (VEGFR1, VEGFR2, and VEGFR3) as well as the coreceptors neuropilin-1 and neuropilin-2 [97]. VEGFR2 is central to angiogenesis, where its activation via dimerization and auto-phosphorylation triggers PLCγ-PKC-Ca^2^⁺ (vascular permeability), PI3K-Akt (cell survival), and Ras-Raf-MEK-ERK (proliferation and migration) pathways [98]. Conversely, VEGFC and VEGFD predominantly bind VEGFR3 to induce lymphatic endothelial proliferation. The ability of these ligands to form VEGFR2 heterodimers allows for critical crosstalk between the vascular and lymphatic systems, facilitating tumor dissemination [99].

The central role of VEGF signaling in various cancers is well-established [100]. In CRC, tumor-derived VEGFA drives abnormal angiogenesis, supporting growth and metastasis [101]. Consequently, anti-VEGF/VEGFR agents are standard treatments for metastatic CRC [102]. In addition to its established kinase activity, VEGFR2 exhibits important nonclassical functions. It acts kinase-independently by recruiting β-arrestin to modulate cell migration and vascular integrity, and it forms trans-family complexes with receptors like fibroblast growth factor receptor (FGFR) and EGFR, increasing pathway diversity and contributing to resistance. VEGFR2 also serves as a central mediator of leukocyte transendothelial migration (TEM), forming a mechanotransduction complex with PECAM-1 and VE-cadherin that initiates TEM via phosphorylation at Y1175, independent of VEGF binding or its intrinsic kinase activity [103]. Endothelial-specific deletion of VEGFR2 blocks neutrophil diapedesis, reducing inflammatory extravasation by ≥ 75% and identifying a novel anti-inflammatory target [104]. This research established that VEGFR2 serves as the core driver of angiogenesis within the context of IBD. Furthermore, inflammation-triggered STAT1 upregulates TGM2, which directly binds and phosphorylates VEGFR2 (Tyr1059/1214), activating pro-angiogenic signaling in IBD. Targeting TGM2 effectively alleviates colitis [105]. Additionally, β-hydroxybutyrate (BHB) reduces tumor burden and angiogenesis in CAC models by inhibiting the hypoxia-inducible factor 1-alpha (HIF-1α)/VEGFA pathway, offering new preventive and treatment strategies [106].

Notably, the VEGF pathway forms a critical intersection with the core pro-survival EGFR/RAS/RAF/MEK/ERK pathway in CRC by activating the downstream Ras-Raf-MEK-ERK cascade. This complexity of the signaling network explains the pleiotropic effects of anti-angiogenic therapies and underscores the importance of the tumor cell’s intrinsic pro-proliferation pathways.

#### EGFR/RAS/RAF/MEK/ERK pathway

The EGFR/RAS/RAF/MEK/ERK signaling cascade is a central oncogenic pathway. It plays a fundamental role in controlling cell proliferation, survival, and differentiation. Aberrant activation of this pathway is a critical driver in the pathogenesis of CRC. The pathway initiates upon extracellular ligand binding (e.g., EGF) to the EGFR tyrosine kinase. Receptor dimerization and autophosphorylation, induced by this event, result in the recruitment of adaptor proteins like Grb2 along with the guanine nucleotide exchange factor SOS. SOS facilitates the exchange of GDP for GTP on membrane-associated G-protein RAS, effectively transitioning RAS from an inactive to an active state, a critical molecular switch [107]. Active RAS-GTP recruits and activates RAF(MAP3K), commencing the core phosphorylation cascade: RAF-MEK (MAP2K)-ERK(MAPK). Upon activation, ERK undergoes nuclear translocation. Within the nucleus, it phosphorylates transcription factors such as c-Fos and c-Myc, that regulate the expression of target genes including Cyclin D1 to drive cell cycle progression. The high mutation frequency of KRAS (∼40%) leads to constitutive RAS activation, persistently driving the downstream RAF-MEK-ERK cascade and promoting cell survival [108]. Similarly, the BRAF V600E mutation (∼8%) causes sustained MEK/ERK phosphorylation, bypassing upstream regulation [109]. These frequent and well-characterized gain-of-function mutations underscore the central role of this pathway and define key therapeutic targets in CRC management.

EGFR constitutes one of the four receptor tyrosine kinases within the ErbB family [110]. This family (ErbB1-4) is frequently overexpressed in CRC and influences numerous tumor-promoting processes through a shared structural architecture [111]. ErbB2 (HER2) is frequently overexpressed in CRC, correlating with poor prognosis and increased tumor aggressiveness, particularly in KRAS G13D mutant contexts [112]. Novel regulatory mechanisms show that the transcription factor ELF3 upregulates KRAS expression under HER2 overexpression, promoting cetuximab resistance [113]. Furthermore, the bacterium *Peptostreptococcus stomati* promotes HER2 activation via its FBA protein binding to integrin α6/β4 on CRC cells, initiating the MEK-ERK-p90 signaling cascade to enhance proliferation, reduce apoptosis, and disrupt intestinal barrier integrity. ErbB3 (HER3), despite having minimal intrinsic kinase activity, functions primarily through heterodimerization (e.g., with HER2), amplifying oncogenic signaling [114]. ErbB4 (HER4), activated by ligands like neuregulins and betacellulin, promotes proliferation and metastasis through the PI3K/Akt and Shc pathways, while simultaneously inhibiting differentiation [115]. The critical nature of this network is confirmed by the observation that loss of HER4 or inhibition of HER3 increases apoptosis, likely by impairing HER3- HER4 heterodimer-dependent Akt signaling [116]. Consistently, ErbB4 knockout reduces intestinal tumor formation in mice, and its suppression sensitizes KRAS-mutant CRC cells to apoptosis [116].

The RAS-RAF-MEK-ERK pathway is a core oncogenic signal downstream of ErbB receptors. EGFR can activate both the RAS-RAF-MEK-ERK and PI3K-AKT-mTOR pathways, but complexes like HER2-HER3 preferentially signal through PI3K-AKT. This differential pathway activation provides the rationale for combining ErbB inhibitors with downstream pathway inhibitors (e.g., RAS-RAF-MEK-ERK) to circumvent resistance mechanisms.

#### PI3K/AKT/mTOR pathway

The PI3K/AKT/mTOR pathway, a frequent activation event in CRC that closely intersects with the EGFR/RAS pathway, is a critical regulator of tumor metabolic reprogramming, angiogenesis, and metastasis. In CRC, this pathway is prominently activated as class I PI3K enzymes convert PIP2 (phosphatidylinositol-4,5-bisphosphate) into the lipid second messenger PIP3 (phosphatidylinositol-3,4,5-trisphosphate). This event recruits and induces Akt phosphorylation, initiating the downstream cascade. Novel regulatory mechanisms have revealed a diverse range of modulators, including coding and noncoding RNA as well as external factors [117]. Recent studies have shown that Proline/Arginine-Rich End Leucine-Rich Repeat Protein (PRELP) exerts a tumor-suppressive effect in CRC [118]. Its expression is significantly downregulated in CRC tissue. Mechanistically, PRELP binds to Fibroblast Growth Factor 1 (FGF1), promoting it degradation. This subsequently inactivates the PI3K/AKT/mTOR pathway, ultimately mitigating tumor angiogenesis and metastasis [119]. Moreover, the exosomal long non-coding RNA MALAT1 drives CRC progression by functioning as a microRNA sponge for miR-26a/26b. This mechanism alleviates the repression of fucosyltransferase 4 (FUT4) and leads to the activation of the PI3K-AKT-mTOR-C-myc signaling axis [120]. Furthermore, the anaerobic bacterium *Solobacterium moorei*, which is enriched in the feces of CRC patients, promotes CRC tumor growth, Its cell wall binds to integrin α2/β1 on CRC cells, thereby activating the PI3K/AKT/mTOR pathway to stimulate tumor growth. Importantly, blocking this specific integrin successfully abolishes the observed pro-tumorigenic effects [121]. Additionally, overexpression of SLIT and NTRK-like family member 4 (SLITRK4) significantly activates the PI3K/AKT pathway, leading to changes in the extracellular matrix and cytokine profile. SLITRK4 promotes infiltration and polarization of TAMs, facilitating CRC occurrence and subsequent liver metastasis. Similarly, LIN28B, overexpressed in approximately 30% of CRC cases, also employs the PI3K/AKT pathway to exacerbate metastatic dissemination to the liver [122].

The PI3K/AKT/mTOR pathway is a central integration hub for signals from diverse upstream pathways, including robust activation by EGFR and transcriptional enhancement by Notch signaling [123, 124]. This cascade is a key mediator of tumor survival and metabolic reprogramming. Its extensive intrapathway crosstalk dictates the need for combined targeted inhibition in clinical trials to block compensatory signaling and overcome therapeutic resistance in clinical trials.

#### JAK/STAT signaling pathway

The JAK/STAT signaling pathway plays a critical role in CRC by integrating signals for cell proliferation, metabolism, inflammation-driven tumorigenesis, and immune evasion [125]. Binding of cytokines and growth factors induces receptor multimerization and activates associated JAK kinases. The JAKs then undergo transphosphorylation and phosphorylate the recruited STAT transcription factors [126]. This mechanism transduces signals directly from the membrane to the nucleus, influencing tumor progression and the tumor microenvironment (TME) [127]. Furthermore, the tissue inhibitor of metalloproteinases-2 (TIMP-2) is markedly upregulated in 5-FU-resistant CRC cells. Research indicates that TIMP-2 specifically promotes resistance to 5-FU chemotherapy by activating the JAK-STAT pathway, establishing a novel mechanism of chemoresistance with potential implications for combination therapies [128].

The JAK/STAT pathway intersects with NF-κB and PI3K/AKT signaling. Proinflammatory cytokines activate both JAK/STAT and NF-κB [129]. Furthermore, PI3K/AKT reinforces the signal by phosphorylating STAT3, enhancing its transcriptional activity. This synergy drives inflammation-driven tumorigenesis and supports combining JAK/STAT inhibitors with anti-inflammatory or PI3K/AKT-targeted drugs in CRC treatment.

#### Notch signaling pathway

The highly conserved Notch signaling pathway is essential for coordinating physiological processes during development and homeostasis, and its dysregulation is pivotal for cell fate determination, stem cell maintenance, and metastasis in CRC [130, 131]. The pathway is built around the Notch transmembrane receptor, which features notch extracellular domain (NECD), transmembrane domain (TMD), and notch intracellular domain (NICD) [132]. Activation occurs via a unique, multistep proteolytic cleavage mechanism: ligand binding (e.g., DLL1) to NECD induces a conformational change and releases the extracellular domain [133]. This critical event allows NICD to be cleaved from the membrane. Once cleaved, NICD translocates into the nucleus, where it functions as a transcriptional co-activator. In the nucleus, its RAM domain mediates binding to the CSL transcription factor. This interaction facilitates the recruitment of co-activators like MAML, leading to the assembly of the transcriptionally active NICD-CSL-MAML ternary complex. This complex then initiates the transcription of effector genes (e.g., Hes and Hey families), thereby regulating core cellular processes critical for CRC progression [134, 135].

Following the comprehensive interpretation of Notch signaling in CRC, novel regulatory mechanisms have emerged [133]. Loss of the glioma tumor suppressor candidate region gene 1 (GLTSCR1) in endothelial cells leads to the upregulation of the Notch ligand JAG1. JAG1 subsequently binds to Notch receptors on CRC cells, activating the pathway and promoting continuous tumor proliferation and metastasis [136]. Furthermore, immune cells within the inflammatory microenvironment activate the PI3K/AKT pathway, which specifically acts on Notch4. This cascade promotes tumor survival and proliferation through the novel Notch4-GATA4-IRG1 axis [137]. Overexpression of the zinc-finger protein 217 (ZNF217) also drives oncogenesis by activating cancer stem cell markers and stimulating the Notch pathway, enhancing the self-renewal capacity of CRC stem cells and facilitating tumor initiation [138]. The functional synergy with the Wnt/β-catenin pathway, where Wnt enhances Notch ligand expression and Notch upregulates β-catenin targets, justifies the combined targeting of these pathways to eliminate cancer stem cells and prevent metastasis.

#### Hippo signaling pathway

The Hippo signaling pathway, which is central to sensing cell contact, mechanical forces, and polarity, is a key regulator of CRC progression and chemoresistance. The core kinase cascade involves MST1/2 kinases activating LATS1/2 and MOB1, leading to the inhibition of the transcriptional co-activator YAP [139, 140]. Furthermore, the Hippo-activated MST1/2 kinases phosphorylate and inhibit PI5P4Ks, which are enzymes that typically suppress MOB1 phosphorylation and LATS activation. The loss of this regulation enhances YAP dephosphorylation and nuclear translocation, thereby promoting proliferation in CRC [141, 142]. Furthermore, *Fusobacterium nucleatum* mediates CRC chemoresistance via the modulation of the Hippo pathway. This microbial action upregulates B-cell lymphoma 2 (BCL2) expression and suppresses chemotherapy-induced, caspase-3/GSDME-mediated pyroptosis, revealing a specific microbial-host signaling interplay that dictates treatment outcome [143].

Resistance to the pan-TEAD inhibitor in CRC involves upregulation of AP-1 and the re-establishment of the YAP- TEAD oncogenic complex [144]. While the inhibitor effectively blocks the initial YAP-TEAD interaction and reduces fos-like antigen 1 (FOSL1) activity, resistant cells overcome this by restoring YAP/TEAD chromatin binding and enhancing MAPK signaling, with FOSL1 being essential for the stabilized YAP-TEAD binding [145, 146]. This unmasked resistance mechanism highlights a critical crosstalk between the Hippo and MAPK pathways in CRC, providing a clear rationale for combining TEAD-targeted therapy with MAPK inhibitors [145]. This approach is further reinforced by the regulatory convergence of the Hippo pathway with MAPK and PI3K/AKT cascades, where MAPK enhances YAP nuclear localization by phosphorylation, while PI3K-AKT promotes YAP activation by inhibiting LATS1/2 [147]. Thus, combining Hippo-targeted therapies with MAPK or PI3K inhibitors is essential for blocking compensatory survival mechanisms.

#### AMP-activated protein kinase pathway

The AMPK pathway represents the core regulatory system for cellular energy homeostasis, playing a key role in metabolic reprogramming and stress adaptation. AMPK coordinates growth and metabolic processes by sensing the AMP/ATP ratio [148]. In CRC, its activation is synergistically regulated by both allosteric modulation and phosphorylation. Under energy stress, AMP/ADP binding to the γ subunit induces allosteric changes that expose the active site, facilitating the phosphorylation of the α subunit at Thr172 by upstream kinases (primarily LKB1 and, in some contexts, CaMKKβ) [149]. Conversely, high ATP levels stabilize the inactive conformation, and phosphatases dephosphorylate Thr172, inhibiting activity. Within the context of metabolic reprogramming, AMPK activation exerts inhibitory effects on lipid metabolism. It phosphorylates key adipogenic enzymes, such as ACC-1, thereby suppressing the expression of FASN, SREBP-1c, and SCD-1, which are essential for lipid biosynthesis and cancer cell proliferation [150]. Additionally, compounds like resveratrol can mitigate the Warburg effect via AMPK activation in CRC models. GLUT3, a key glucose transporter involved in metabolic regulation, is highly expressed in CRC, where its overexpression is associated with poor prognosis, further highlighting its dual role in metabolic control. Under glucose-deficient conditions, GLUT3 promotes CRC progression by enhancing glucose uptake and promoting nucleotide synthesis. This process is critically modulated by the AMPK/CREB1 pathway, which markedly upregulates GLUT3 expression in response to low-glucose stress [151].

Signaling pathways operate as a complex, cross-regulatory network that collectively drives colorectal carcinogenesis. The AMPK pathway acts as a central metabolic regulator, interacting with oncogenic cascades [152]. This pathway negatively regulates mTOR signaling downstream of PI3K/AKT, modulates Wnt/β-catenin activity via phosphorylation, and affects Hippo effectors through energy sensing [153, 154]. Similarly, the EGFR/RAS/RAF/MEK/ERK axis intersects with VEGF signaling in angiogenesis, while the JAK/STAT and Notch pathways promote inflammation-driven progression [155, 156]. This extensive network of pathway crosstalk helps explain the limited efficacy of single-pathway targeted therapies and highlights the need for multitarget strategies that simultaneously inhibit compensatory signaling mechanisms and overcome drug resistance [157].

The development of targeted therapies for CRC is directly propelled by its sophisticated molecular pathogenesis, which involves complex interactions between signaling pathways. Concurrently, deciphering the critical molecular nodes within these pathways provides both a theoretical foundation and actionable targets for such therapeutic strategies.

## Targeted therapies in colorectal cancer

Based on the molecular pathogenesis of CRC, targeted therapy has emerged as a key approach for precision treatment, specifically blocking critical carcinogenic pathways. This approach enables precise tumor eradication by selectively disrupting oncogenic signaling and apoptotic pathways, thereby minimizing toxicity to normal tissues [158, 159]. Rapid advances in gene sequencing have led to the identification of numerous key targets, prompting the development of a therapeutic arsenal that includes small-molecule inhibitors and monoclonal antibodies [160]. The mechanisms, clinical applications, and future trajectories of these key targets and their corresponding agents will be systematically explored in the following sections.

### Targeting Wnt/β-catenin signaling pathway

The Wnt/β-catenin pathway is a key oncogenic driver in most CRCs yet it remains an “undruggable” target with no approved direct inhibitors, due to β-catenin's lack of druggable pockets, its vital role in gut stem cells (narrowing the therapeutic index), and feedback resistance mechanisms [161, 162].

Consequently, the therapeutic focus has shifted to indirect strategies, which can be categorized by developmental stage. Agents under clinical investigation: PORCN inhibitors (e.g., LGK974, CGX1321) block Wnt ligand secretion and have shown early proof-of-mechanism, but their development is hampered by dose-limiting toxicities like bone disorders [163, 164]. Their application is now focused on biomarker-selected patients (e.g., RNF43 mutations) [165, 166]. Another approach is targeting the β-catenin transcriptional complex with agents like PRI-724 or E7386, which aim to disrupt oncogenic transcription and are in early clinical evaluation [167]. Preclinical and repurposing candidates include strategies to restore β-catenin degradation. Tankyrase inhibitors (e.g., XAV-939) stabilize AXIN but cause on-target toxicity [168], while the repurposed drug pyrvinium acts as a CK1α agonist to enhance β-catenin destruction [169]. Novel modalities such as PROTACs and synthetic lethal strategies targeting coactivators like BCL9 are also under exploration [166]. Key translational hurdles are on-target toxicities, pathway complexity, and a lack of biomarkers [170]. Overcoming these will require innovative delivery systems and rational combinations to finally integrate Wnt inhibition into CRC precision medicine [171, 172].

### Targeting VEGF pathway inhibitors

As a primary regulator of pathological angiogenesis, VEGF signaling is central to CRC proliferation and metastasis, validating antiangiogenic therapy as a core treatment strategy [173].

Clinically approved agents: current VEGFR-targeting agents are classified based on their mechanism of action. Monoclonal antibodies, such as bevacizumab, act extracellularly by sequestering the VEGF ligand, thereby preventing its binding to VEGFR and inhibiting downstream signaling [174]. Although this approach confers a survival benefit, long-term treatment is associated with the development of drug resistance, often mediated by the upregulation of alternative proangiogenic factors, and clinically challenging systemic adverse effects, including hypertension and proteinuria. The second class, small-molecule inhibitors such as regorafenib are multikinase inhibitors that act intracellularly. Pharmacologically, these agents are limited by poor selectivity, which contributes to dose-limiting off-target toxicities and restricts drug exposure at the maximum tolerated dose, resulting in insufficient and transient VEGFR inhibition [175]. Collectively, the limitations of both established and newer agents such as fruquintinib [176], anlotinib [177], and faricimab [178], including acquired resistance and systemic toxicity, highlight the urgent need for the continued development of novel VEGFR-targeted therapies that provide safer and more durable therapeutic outcomes for patients with CRC.

Agents under clinical investigation: Brivanib (BMS-582664), an orally administered tyrosine-kinase inhibitor (TKI), selectively targets FGFR and VEGFR, thereby blocking signaling pathways essential for tumor angiogenesis and proliferation. Preclinically, brivanib significantly inhibited tumor growth, reduced microvascular density, and increased apoptosis in hepatocellular carcinoma (HCC) xenograft models [179]. However, clinical observations from brivanib-cetuximab treatment in mCRC showed that treatment-induced hypertension (TI-HTN) was not related to patient response or survival (P > 0.05). Metabolomic analysis identified 29 TI-HTN-related metabolites associated with vasomotor dysregulation, with overlap to preeclampsia signatures; however, the lack of a survival benefit indicates that TI-HTN should be excluded as a surrogate for therapeutic efficacy, limiting its relevance to investigations of toxicity mechanisms [180]. Nintedanib alone or in combination with capecitabine in refractory mCRC (NCT02780700).

Experiments in cell culture and tumor xenograft models have shown that fruquintinib synergizes effectively with chemotherapeutic agents such as doxorubicin and oxaliplatin, resulting in an approximately 30% increase in tumor growth inhibition rate [181]. Notably, at lower doses, fruquintinib enhances antitumor immune responses when combined with anti-PD-1 antibodies. This immunomodulatory effect is characterized by reduced angiogenesis, increased migration of CD8 + T cells, and decreased proportions of immunosuppressive cells such as macrophages and MDSCs, thereby remodeling the tumor immune microenvironment [182]. Clinical trials targeting this specific molecular pathway include four completed studies (NCT02009449, NCT00640471, NCT02870582, and NCT02390947), although detailed results have not yet been published. Additional trials are currently in development (Table 1).

**Table 1:** Agents targeting VEGFR under clinical investigation

Drug	Targets	Phase	Tumor type	Trial name	Enrollment	Primary outcome	Stutas
Cediranib	VEGFR2/VEGFR3	II	Advanced solid tumors	NCT03851614	90	Changes in genomic and immune biomarkers	Active
Sunitinib	VEGFR/PDGFR	II	CRC/mCRC	NCT02465060/ NCT00961571	6452 50	ORR PFS	Active Terminated
Pazopanib	VEGFR/PDGFR/FGFR	I	Advanced solid tumors	NCT02009449	353	AEs	Completed
Nintedanib	VEGFR/PDGFR/FGFR	II	mCRC	NCT02780700	1	PFS	Terminated
FPI-2053	VEGFR/PDGFR/FGFR	I	Advanced solid tumors	NCT06147037	70	AE, DLT, RAD, RP2D	Recruiting
Brivanib	VEGFR/FGFR	III	mCRC	NCT00640471	750	OS	Completed
Donafenib	VEGFR/PDGFR/RAF	III	mCRC	NCT02870582	536	OS	Completed
Olinvacimab	VEGFR	Ib/​ II	mCRC	NCT04751955	0	MTD, PFS	Withdrawn
KL-140	EGFR	II	mCRC Ras wild-type	NCT03290937	34	RP2D, ORR	Completed
Famitinib	PDGFR/VEGFR	III	Advanced CRC	NCT02390947	543	OS	Completed
Fruquintinib	VEGFR	II	mCRC	NCT07051785	68	PFS	Not yet recruiting
TAS-115	VEGFR/c-Met	I	CRC	NCT04999761	917	DLT, ORR, PFS	Recruiting
XL092	VEGFR/PDGFR/FGFR	I III	Solid tumors and mCRC	NCT03845166/NCT05425940	325,901	MTD, ORR, PFS, OS OS, NLM	Active

Data source: Clinical data obtained from https://clinicaltrials.gov

### Targeting the EGFR/RAS/RAF/MEK/ERK pathway

In addition to anti-angiogenesis, the EGFR/RAS/RAF/MEK/ERK pathway is a major target. Its abnormal activation is central to CRC carcinogenesis and progression. The well-defined mutation status of key signaling nodes provides an explicit molecular basis for therapeutic selection, offering a clear roadmap for targeted intervention strategies in this disease.

#### Targeting the EGFR family

Targeting the EGFR pathway is a crucial approach for inhibiting CRC proliferation and survival, considering the strong association of abnormal EGFR activation with tumor cell proliferation and survival. The efficacy of anti-EGFR drugs is intrinsically associated with the mutation status of downstream components, confirming that molecular profiling is critical for treatment selection.

Clinically approved agents: The primary approved anti-EGFR agents are monoclonal antibodies, cetuximab and panitumumab, which act by binding to the EGFR extracellular domain [183]. This binding inhibits ligand-induced dimerization and autophosphorylation, suppressing the downstream RAS-RAF-MEK-ERK and PI3K-AKT-mTOR signaling pathways [184]. Their therapeutic efficacy is strictly dependent on the mutation status of downstream effectors. These agents are effective only in mCRC patients with wild-type RAS/BRAF. KRAS mutations, occurring in 40%−50% of patients, constitutively activate the MAPK/PI3K axis, bypassing upstream EGFR blockade and representing a major mechanism of intrinsic resistance [185]. The high incidence of primary and acquired resistance substantially limits the clinical benefit of anti-EGFR monotherapy. Therefore, continuous research is focused on developing agents with additional, secondary targets to circumvent this resistance and enhance therapeutic outcomes (Table 2).

**Table 2:** Agents targeting EGFR family under clinical investigation

Drug	Targets	Phase	Tumor type	Trial name	Enrollment	Primary outcome	Stutas
BAY2927088	EGFR/HER2	II	HER2 mutant solid tumor	NCT06760819	111	ORR	Recruiting
SI-B001	EGFR/HER3	II	CRC	NCT05039944	7	ORR; Optimal combination dose (only IIa)	Terminated
MCLA-129	EGFR/c-Met	I/II I/II	Solid tumors	NCT04930432 NCT04868877	400 576	DLT, MTD, ORR, TEAE, MTD, RP2D, ORR	Recruiting Recruiting
HLX07	EGFR	II	mCRC	NCT05239650	50	ORR, PFS	Not yet recruiting
A166	HER2	I/II	CRC	NCT03602079	49	MTD	Completed
ACE1702	HER2	I	HER2 positive solid tumors	NCT04319757	12	AEs, DLTs, SAEs	Completed
ARX788	HER2	II	HER2 positive solid tumors	NCT05041972	0	ORR	Withdrawn
Sevabertinib	EGFR/HER2	II	Solid tumors	NCT06760819	111	ORR	Recruiting
BDC-1001	HER2	I/II	HER2 positive solid tumors	NCT04278144	175	AEs, SAEs, DLTs, MTD, ORR, CR, PR	Terminated
BL-M17D1	HER2	I	HER2 positive CRC	NCT06500052	20	DLT, MTD, RP2D	Recruiting
Disitamab vedotin	HER2	IIII	HER2 positive mCRC	NCT05661357	51	ORR	Active
ELVN-002	HER2	I	HER2 positive solid tumors/CRC	NCT06328738	275	DLTs, AEs	Active
SPH5030	HER2	II	HER2 positive/mutant CRC	NCT06434597	60	ORR	Recruiting
Pertuzumab	HER2	II	mCRC	NCT05725200	40	TEAEs, ECG AEs	Recruiting
KJ015	HER2	I	HER2 positive solid tumors	NCT07036185	66	DLTs, MTD, RED, AE	Not yet recruiting
KN026	HER2	II	HER2 positive CRC	NCT05985707	80	ORR	Not yet recruiting
IAM1363	HER2	I	HER2 positive cancers	NCT06253871	243	DLTs, AEs, ORR	Recruiting
XMT-2056	HER2	I	HER2 positive solid tumors	NCT05514717	162	DLTs, AEs, ORR	Recruiting
ZW25	HER2	II	CRC	NCT03929666	74	DLTs, AESI,ORR	Completed

Data source: Clinical data obtained from https://clinicaltrials.gov

Agents under clinical investigation: To overcome the limitations of single-target inhibition, a new generation of agents is under investigation, categorized as follows:1) Novel TKIs: BAY 2927088 is a selective compound with a low molecular weight TKI with a dual-target mechanism, and inhibits dihydroorotate dehydrogenase (DHODH). It has shown promising initial efficacy in non-small cell lung cancer (NSCLC) and is under evaluation in clinical trials for CRC [186]. 2) Bispecific antibodies: These are designed to overcome resistance from pathway bypass. SI-B001, an EGFR × HER3 bispecific antibody, simultaneously inhibits EGFR homodimers and EGFR/HER3 heterodimers, aiming to preemptively shut down downstream RAS-RAF-MEK-ERK and PI3K-AKT-mTOR signaling to overcome bypass resistance, thereby countering bypass-mediated resistance [187]. Another key agent, MCLA-129, is a bispecific antibody that targets both EGFR and the mesenchymal-epithelial transition factor (c-Met) receptor. By blocking these two oncogenic pathways, MCLA-129 suppresses tumor growth and enhances host immunity by boosting antibody-dependent cell-mediated cytotoxicity, thereby promoting cancer cell destruction [188]. 3) Biosimilars and structurally optimized antibodies**:** QL1203, a recombinant fully human anti-EGFR monoclonal antibody injection, serves as a biosimilar to panitumumab (Vectibix). In Phase III clinical trials for first-line therapy of RAS wild-type mCRC, QL1203 demonstrated efficacy and safety profiles comparable to the reference product. Considering this clinical equivalence, QL1203 is anticipated to be an accessible and cost-effective alternative to established EGFR-targeted monoclonal antibody therapy, particularly in regions such as China [189]. Furthermore, structurally optimized agents such as humanized anti-EGFR monoclonal antibody (HLX07) are being developed to improve affinity and reduce immunogenicity compared to cetuximab. Currently, this drug is undergoing phase II clinical trials for mCRC [190].

HER2 is an oncogenic member of the ErbB receptor family. Targeting HER2 with specific antibodies or antibody- drug conjugates (ADCs) represent a highly effective therapeutic strategy for approximately 3% of patients with CRC and HER2 overexpression or amplification, a subgroup generally resistant to conventional chemotherapy [191]. HER2 has been formally validated as a therapeutically actionable driver biomarker in CRC, based on compelling data from HERACLES, MyPathway, MOUNTAINEER, and DESTINY-CRC01 studies [192].

Clinically approved agents: The combination of the monoclonal antibodies trastuzumab and pertuzumab is an established regimen. Anti-HER2 agents such as trastuzumab selectively bind to HER2, disrupting downstream signaling to inhibit tumor growth and proliferation. Beyond monoclonal antibodies, novel modalities are being investigated [193, 194]. ADCs are advanced sophisticated biopharmaceuticals consisting of monoclonal antibodies conjugated via synthetic linkers to potent cytotoxic drugs. They facilitate precise delivery of chemotherapy to HER2-expressing tumors, maximizing therapeutic effect and not affecting healthy tissues [195]. The combination of anti-HER2 monoclonal antibodies, trastuzumab and pertuzumab, with ADC trastuzumab deruxtecan (T-DXd), offers a promising new therapeutic option [196]. T-DXd has demonstrated exceptional efficacy in the DESTINY-CRC01 study, achieving an ORR of 45% and a median PFS of 8.2 months [196]. Notably, T-DXd maintained efficacy despite the presence of RAS/RAF mutations, which typically confer resistance to upstream targets. This finding suggests a potential paradigm shift in the treatment of HER2-positive CRC [197].

Agents under clinical investigation: Several next-generation HER2-targeted ADCs are currently under development. This includes novel ADCs such as A166, Phase I studies have demonstrated a manageable safety profile, favorable pharmacokinetics, and promising initial antitumor activity [198]. The novel antibody–drug conjugate ARX788 showed superior efficacy over prior treatments, significantly increasing both median progression-free survival and objective response rates in pretreated patients with HER2-positive advanced breast cancer. It is currently under investigation in multiple clinical studies targeting HER2-expressing advanced solid tumors, including CRC. The phase I trials have been completed [199]. The emerging anti-HER2 therapeutic landscape includes ACE1702, an off-the-shelf antibody- conjugated NK cell therapy. This cellular agent is currently undergoing evaluation in the dose-escalation phase of a phase I clinical trial (NCT04319757) for HER2-expressing advanced or metastatic solid tumors, including CRC [200]. In addition, the small-molecule inhibitor sevabertinib (BAY-2927088), an oral, reversible, mutation-selective dual EGFR/HER2 TKI, provides a targeted strategy that effectively addresses specific mutations. This drug has progressed rapidly and is currently undergoing global phase II clinical trials for CRC and other HER2-mutated solid tumors [201].AZD8931, a small-molecule TKI targeting EGFR, HER2, and HER3, demonstrated good tolerability and preliminary efficacy in its phase I/II trial when combined with FOLFIRI in mCRC, and has since progressed to phase II [114]. (Table 2).

A phase II study (NCT0632873) evaluating the bispecific antibody zanidatamab (ZW25) plus chemotherapy in HER2-expressing advanced gastroesophageal adenocarcinoma, biliary tract cancer, and CRC is among three recently completed trials. The study revealed potent first-line efficacy for ZW25 in HER2-positive advanced gastroesophageal adenocarcinoma, with a confirmed ORR of 76.2%, a median OS of 36.5 months, and a PFS of 12.5 months. The regimen maintained a manageable safety profile, with diarrhea as the main adverse event, which was significantly mitigated by prophylactic medication. These robust outcomes suggest that ZW25 plus chemotherapy may emerge as the new first-line standard of care for HER2-positive advanced gastroesophageal adenocarcinoma [202]. Data from the two remaining trials are currently pending release.

#### Targeting KRAS and BRAF

KRAS and BRAF mutations are key oncogenic drivers, resulting in constitutive pathway activation and autonomous cell growth. Therapeutic targeting of key pathway nodes is a highly rational approach for tumors harboring these specific mutations [203]. To present the drug development landscape more clearly, targeted agents are discussed below according to their stage of development.

Clinically approved therapies: Targeting BRAF mutations in CRC is challenging due to inherent resistance mechanisms. Monotherapy with BRAF inhibitors (e.g., vemurafenib) has shown poor response rates (< 5%) because of the rapid and compensatory EGFR feedback loop that swiftly reactivates the MAPK pathway [204]. This necessity for overcoming feedback and chemoresistance led to the adoption of encorafenib plus cetuximab combined with mFOLFOX6 as the first-line standard of care for BRAF V600E-mutant mCRC, with the doublet used for second-line therapy [205].

Agents under clinical investigation: Several drugs targeting mutant KRAS and BRAF are in active clinical trials. KRAS-Targeted Agents: Sotorasib, the first KRAS G12C inhibitor approved by the U.S. FDA for metastatic NSCLC, showed a modest ORR of only 9.7% in CRC [206]. Currently, there are several drugs targeting the KRAS oncogene undergoing clinical trials, including inhibitors targeting mutations such as KRAS G12C and G12D [25, 204]. The KRAS G12C selective covalent inhibitor adagrasib (MRTX849) is a promising alternative, having started clinical evaluation in 2018 through the KRYSTAL-1 study (NCT03785249) for advanced solid tumors. Adagrasib has progressed to phase III clinical trials (NCT04793958) for patients with KRAS G12C-mutated CRC. Multiple novel agents targeting the KRAS oncogene are currently in clinical trials (Table 3). BRAF-Targeted Agents: BRAF-mutant CRC exhibits resistance to standard chemotherapeutic drugs such as irinotecan [207]. Novel BRAF-targeted drugs and optimized combinations are needed to improve patient outcomes. The next-generation pipeline includes several candidates, such as naporafenib, a dual BRAF/CRAF kinase inhibitor currently being assessed in CRC after demonstrating promising results in trials.

**Table 3:** Agents targeting KRAS and BRAF under clinical investigation

Drug	Targets	Phase	Tumor type	Trial name	Enrollment	Primary outcome	Stutas
Adagrasib	KRAS G12C	III	CRC (KRAS G12C)	NCT04793958	461	OS, PFS	Active
AFNT-211	KRAS G12V	I/II	CRC	NCT06105021	100	OBD, RP2D, AEs, DLT, TEAEs	Active
ALTA3263	KRAS	I	CRC (KRAS mutant)	NCT06835569	188	TEAEs, DLTs	Recruiting
ARV-806	KRAS G12D	I/II	CRC (KRAS G12D)	NCT07023731	159	AEs, ORR	Recruiting
ASP4396	KRAS G12D	I	CRC	NCT06364696	175	DLTs, AEs, SAEs, ECG	Recruiting
BBO-11818	KRAS G12C/D/V	I	Cancer (KRAS mutant)	NCT06917079	387	TEAEs, SAEs, DLTs	Recruiting
BGB-53038	KRAS G12C/D	I	Metastatic solid tumors (KRAS mutant)	NCT06585488	514	AEs, MTD, MAD, ORR, RP2D	Recruiting
ELI-002 2P	KRA G12	I	Solid tumors (KRAS mutant)	NCT04853017	25	TEAEs,	Completed
ELI-002 7P	KRA GS	I/II	Solid tumors (KRAS/NRAS mutant)	NCT05726864	158	AEs, DFS	Active
QLC1101	KRAS G12	I	Advanced solid tumors	NCT06403735	250	DLT, MTD (or MAD), RP2D	Recruiting
MRTX0902	SOS1/KRAS	I/II	CRC (KRAS G12C)	NCT05578092	228	ORR, DOR, PFS, OS	Active
MRTX1133	KRAS G12D	I	CRC (KRAS G12C)	NCT05737706	63	DLTs, TEAEs, ORR, DOR, PFS, OS	Terminated
LY3962673	KRAS G12D	I	CRC (KRAS G12D)	NCT06586515	630	TEAEs, AEs, DLT, ORR, BOR, DOR, TTR, DCR	Recruiting
IK-595	MEK-ERK	I	RAS or RAF altered advanced CRC	NCT06270082	75	RP2D, TEAEs, DLT,	Terminated
Inavolisib	KRAS G12C	I	mCRC	NCT04929223	542	ORR	Recruiting
Divarasib	KRAS G12C	I	mCRC	NCT04929223	542	ORR	Recruiting
INCB161734	KRAS G12D	I	CRC (KRAS G12C)	NCT06179160	710	DLTs, TEAEs	Recruiting
INCB186748	KRAS G12D	I	CRC (KRAS G12C)	NCT06818812	30	DLTs, TEAEs	Active
HBI-2438	KRAS G12C	I	CRC (KRAS G12C)	NCT05485974	44	MTD, DLTs, AEs	Active
GDC-6036	KRAS G12C	I I	CRC (KRAS G12C) mCRC	NCT04449874/ NCT04929223	498 542	AEs, DLTs ORR	Active Recruiting
FMC-376	KRAS G12C	I/II	CRC (KRAS G12C)	NCT06244771	403	DLTs, AEs, TEAEs	Recruiting
VS-7375	KRAS G12D	I/II	CRC (KRAS G12D)	NCT07020221	330	AEs, TEAEs, TRAEs, SAEs, DLTs, MTD	Recruiting
ABM-1310	BRAF V600E	I	Advanced solid tumors	NCT04190628	53	MTD, RP2D,	Terminated
BGB-3245	BRAF/CRAF/ARAF	I I	Advanced or metastatic RAS mutant CRC	NCT06194877 NCT05907304	13 86	SAEs, TEAEs, AESIs, MTD, RP2D, ORR ORR	Terminated Active
LUT014 Gel (Dose 1)	BRAF	II	mCRC	NCT04759664	117	TSR	Unknown status
LXH254	BRAF/CRAF/ARAF	I	Advanced or metastatic solid tumors	NCT05907304	86	ORR	Active
HLX208	BRAF V600E	II	mCRC (BRAF V600E)	NCT04984369	50	ORR	Unknown status
LGX818	BRAF V600E	I I/II II	melanoma and mCRC mCRC (BRAF V600E) CRC	NCT01436656/ NCT04017650/ NCT06640166	107 38 25	DLT, AE, ALT, AST AE 6-month PFS	Completed Active Recruiting

Data source: Clinical data obtained from https://clinicaltrials.gov

KRAS- or BRAF-mutant NSCLC and NRAS-mutant melanoma. A highly promising new agent is BGB-3245, a selective oral small-molecule BRAF inhibitor [208]. BGB-3245 exhibits broad-spectrum activity, targeting BRAF V600 and non-V600 mutations, as well as RAF fusion proteins. Preclinical studies have demonstrated its capacity to inhibit both monomeric and dimeric BRAF species, thereby reducing the dimer-driven resistance commonly observed with earlier inhibitors [209]. The clinical development of BGB-3245 began with a phase I study (NCT04249843) initiated in February 2020. Enrollment data up to 2022 confirmed that 42 patients were treated, with results demonstrating a manageable safety profile for the drug. Moreover, a phase I/IIa combination study is currently underway, investigating the therapeutic potential of BGB-3245 in combination with mirdametinib (NCT05580770). These initiatives are part of a broader, active clinical landscape featuring several additional BRAF inhibitors (Table 3).

Two completed or ongoing early-phase clinical trials have shown novel approaches in solid tumor treatment. The lead trial, ELI-002 2P (NCT04853017), is a first-in-human phase I study evaluating the safety, tolerability, and immunogenicity of a novel immunotherapy in the minimal residual disease setting for patients with KRAS/NRAS-mutated pancreatic or CRC. This study focuses on dose escalation to determine the appropriate phase II dose. Conversely, the trial LGX818 (NCT01436656) reported a preliminary efficacy signal in a subset of mCRC patients, reporting an OS of 4.5 months and 4.0 months in the 300 mg and 400 mg dose groups, respectively. These studies collectively map the current trajectory of clinical development, spanning dose-finding, initial efficacy signal reading, and pending data disclosure for targeted and immunotherapies in solid tumors.

#### Targeting MEK and ERK

As a critical kinase within the RAS/RAF/MEK/ERK signaling pathway [210], MEK is a major therapeutic target. MEK inhibitors such as trametinib and cobimetinib are commonly used in combination regimens to achieve enhanced antitumor effects [211, 212]. Clinically approved therapies**:** Trametinib, a highly selective MEK1/2 inhibitor approved by the FDA, is the established partner for Dabrafenib in treating BRAF V600E-mutant metastatic melanoma [213]. In CRC, inhibiting MEK effectively blocks the RAS-RAF-MEK-ERK cascade, playing a crucial role in suppressing tumor-cell proliferation and survival. Cobimetinib and binimetinib, which are oral, non-ATP-competitive MEK1/2 inhibitors also approved by the FDA, similarly disrupt the RAS-RAF-MEK-ERK pathway in CRC [214, 215]. These inhibitors have demonstrated substantial clinical efficacy in various cancers, especially when strategically combined with BRAF inhibitors to overcome feedback activation [216]. Agents Under Clinical Investigation**:** Advancing through the clinical pipeline, the next-generation agent avutometinib is under investigation in a phase III trial in combination with defactinib for recurrent low-grade serous ovarian cancer. Additionally, a separate phase II clinical study is evaluating avutometinib in CRC [217]. The full spectrum of ongoing MEK-targeted clinical trials is summarized in Table 4.

**Table 4:** Agents targeting MEK and ERK under clinical investigation

Drug	Targets	Phase	Tumor type	Trial name	Enrollment	Primary outcome	Stutas
ABM-168	MEK1/2	I	Advanced solid tumors	NCT05831995	12	DLT, AEs	Terminated
(Binimetinib)		I/II II	mCRC Refractory CRC Advanced refractory solid tumors	NCT03374254 NCT03475004 NCT02465060	116 53 6452	DLT ORR ORR	Completed Completed Active
Avutometinib	RAF/MEK	II	Anti-EGFR refractory advanced CRC	NCT06369259	33	AEs	Recruiting
Nivolumab	MEK1/2	II	CRC	NCT02060188	385	ORR, CR, PR	Completed
Selumetinib	MEK1/2	I	Advanced solid tumors or advanced or mCRC	NCT02188264	40	DLT	Completed
IDE196	PKC	I/II	Solid tumors	NCT03947385	341	DLT, MTD, RP2D, ORR	Recruiting
IK-595	MEK-RAF	I	RAS or RAF altered advanced tumors	NCT06270082	75	DLT, AEs, RP2D	Terminated
HL-085 (Tunlametinib)	MEK1/2	II	mCRC	NCT05233332	186	ORR	Unknown status
	MEK1/2	III	mCRC (BRAFV600E)	NCT06008119	165	PFS	Recruiting
VS-6766	RAF/MEK	I/II	Advanced CRC	NCT05200442	53	MTD, ORR	Recruiting
LY3214996	ERK1/2	I I/II	mCRC (KRAS mutant), mCRC	NCT04916236 NCT04616183	24 46	MTD, CR, PR	Terminated Active
IPN01194	ERK1/2	I/II	CRC	NCT06305247	220	DLT, TEAEs, ORR	Recruiting
Ulixertinib	ERK1/2	I	mCRC	NCT05985954	27	AEs	Recruiting
MK-8353	ERK1/2	I	CRC	NCT02972034	111	AEs	Terminated

Data source: Clinical data obtained from https://clinicaltrials.gov

ERK serves as a key downstream factor in the RAS-RAF-MEK-ERK signaling pathway, with its overactivation linked to CRC proliferation, survival, and drug resistance [210]. ERK activation drives tumorigenesis, and its inhibition effectively suppresses tumor cell viability [218, 219]. Agents Under Clinical Investigation: Ulixertinib (BVD-523) is an oral, reversible, ATP-competitive inhibitor of ERK1/2 that blocks downstream MAPK signaling by suppressing catalytic activity [220]. Phase I trial results indicated that the ERK1/2 inhibitor ulixertinib had a defined recommended dose of 600 mg twice daily. The agent exhibited a favorable safety profile and displayed preliminary antitumor activity in patients with advanced solid tumors harboring NRAS or BRAF mutations [221]. Additionally, IPN01194 is a first-in-class, oral small-molecule ERK1/ERK2 inhibitor, currently in phase I/IIa clinical development, this agent targets the downstream nodes of the MAPK pathway to block aberrant proliferative signals. MK-8353 is another orally bioavailable, ATP-competitive small-molecule ERK1/2 inhibitor. Its phase Ib trial (NCT03745989) assessed the combination of MK-8353 with the MEK inhibitor selumetinib in advanced solid tumors, focusing on safety, tolerability, and defining the recommended phase II dose [222].

Preclinical/Experimental: LY3214996 (temuterkib) is an orally available ERK1/2 inhibitor that acts as an ATP-competitive antagonist, thereby blocking the kinase activity of both ERK1 and ERK2 to suppress the downstream MAPK cascade. By suppressing the phosphorylation of substrates (pRSK1, p–c-MYC, and Cyclin D1), LY3214996 promotes G1-phase arrest and apoptosis. Notably, this compound is capable of overcoming resistance mediated by ERK reactivation, which is commonly observed with upstream BRAF or MEK inhibitors [223, 224]. The CheckMate 142 trial (NCT02060188) confirmed the robust efficacy of nivolumab plus ipilimumab in recurrent/metastatic MSI-H CRC, achieving a high ORR (71.1%) compared with monotherapy (40.5%). This efficacy was not replicated in patients with non-MSI-H/MS, or in certain combination regimens (such as nivolumab plus daratumumab), indicating a strong association between MSI status and therapeutic response (Table 4).

### Targeting the PI3K/AKT/mTOR Pathway and SHP2

The PI3K/AKT/mTOR signaling pathway is crucial for CRC initiation, progression, and metastasis, with its dysregulation strongly correlated with tumor invasiveness and drug resistance. Because of this central oncogenic role, PI3K-targeted therapies are highly anticipated [123]. The following presents PI3K/AKT/mTOR-targeted agents categorized by their developmental stage.

Clinically approved agents: inhibitors such everolimus successfully suppresses this pathway and inhibit tumor-cell proliferation [225]. This drug class for treating other cancers has been approved by the FDA, including idelalisib (PI3Kδ), copanlisib (pan-PI3K), duvelisib (PI3Kδ/γ), and alpelisib (PI3Kα) [226–228]. Agents Under Clinical Investigation: The path to achieving efficacy in CRC is complex. Earlier broad-spectrum agents, such as the pan-PI3K inhibitor PX-866, failed to improve patient outcomes in combination therapies and instead increased treatment-related toxicity. This outcome reflects the problem of limited specificity, as PI3K inhibition in normal cells causes dose-limiting adverse effects [229]. The novel small-molecule organic heterocyclic drug BBO-10203 (Bridgebio Pharma Inc.) specifically block the oncogenic RAS-PI3Kα interaction, thereby restricting tumor proliferation without the systemic effect of hyperglycemia [230]. Preclinical xenograft models have demonstrated its efficacy and a favorable low toxicity profile, leading to its current advancement in clinical trials. The PI3K pathway remains a focal point in oncology, with other inhibitors, such as parsaclisib (PI3Kδ inhibitor) for B-cell malignancies, in active clinical evaluation [231].

Moreover, compensatory signaling mechanisms within tumor cells can further weaken therapeutic benefits. Consequently, the development of highly selective next-generation PI3K-targeted agents and the strategic exploration of effective combination regimens are key directions for advancing CRC treatment. The complete list of agents targeting this pathway is summarized in Table 5.

**Table 5:** Agents targeting the PI3K/AKT/mTOR pathway, SHP2 and PARP under clinical investigation

Drug	Targets	Phase	Tumor type	Trial name	Enrollment	Primary outcome	Stutas
BBO-10203	RAS/PI3Kα	I	mCRC (KRAS)	NCT06625775	392	MTD, TEAEs, DLTs	Recruiting
BKM120	PI3K	I/II	Metastatic/advanced RAS wild type CRC	NCT01591421	22	AEs, TD, DLTs	Completed
BYL719	PI3Kα	I/II	mCRC (PIK3CA mutant)	NCT04753203	65	MTD, PFS	Unknown status
CVL237	PI3K-p110β/δ	II	Advanced solid tumors	NCT06183736	98	ORR	Not yet recruiting
DKN-01	DKK1	II	CRC	NCT05480306	221	PFS	Completed
Savolitinib	c-Met	II	CRC (MET amplified metastatic or unresectable)	NCT03592641	5	ORR	Terminated
MEN1611	PI3K	I/II	mCRC	NCT04495621	29	RP2D, ORR, DLT	Completed
KO-2806	FTI	I	Advanced solid tumors	NCT06026410	300	DLTs, AEs, ORR	Recruiting
Idelalisib	PI3Kδ	II/I	mCRC Metastatic solid tumors (KRAS G12C)	NCT05725200/NCT04449874	40 498	ORR AEs, DLTs	Recruiting Active
INCB050465	PI3Kδ	I	CRC	NCT02646748	159	AEs	Completed
Nab- Rapamycin	mTOR	I/II	mCRC	NCT03439462	60	DLTs	Completed
BMS-986466	SHP2	I/II	Solid tumors (KRAS-G12C)	NCT06024174	5	DLTs, AEs, SAEs, ORR	Terminated
ERAS-601	SHP2	I	Advanced or metastatic solid tumors	NCT04670679	200	DLT, MTD, RD, AEs, Cmax, Tmax	Active
HBI-2376	SHP2	I	KRAS of EGFR mutant solid tumors	NCT05163028	42	MTD, RP2D, DLTs, AEs, SAEs	Active
Tinodasertib	SHP2	II	mCRC	NCT05462236	120	AEs, DLT, TEAEs, ORR	Recruiting
TNO155	SHP 2	I	CRC	NCT04000529/NCT04330664	122 86	DLT, AEs AEs	Terminated Completed
RMC-4630	SHP2	I	Metastatic cancers (KRAS mutant)	NCT04916236	24	MTD	Terminated
JAB-3068	SHP2	I/II	Advanced solid tumors	NCT03565003	126	DLT, ORR, DOR	Completed
JAB-3312	SHP2	I/II	Advanced solid tumors (KRAS G12C)	NCT05288205	240	RP2D, DLT	Recruiting
VB15010 (Olaparib)	PARP	III/II II	mCRC CRC	NCT04456699/NCT04166435/	335 11	PFS ORR	Completed Completed
		I/II	Advanced solid tumors	NCT06819215	188	AEs	Recruiting
Niraparib	PARP	II	Advanced or mCRC	NCT03983993	26	CBR	Active
Rucaparib	PARP	I/II	CRC	NCT03337087	18	DLT, MTD	Active

Data source: Clinical data obtained from https://clinicaltrials.gov

The nonreceptor protein tyrosine phosphatase Src-homology-2 domain-containing phosphatase 2 (SHP-2) is a key signaling node that modulates multiple downstream cascades, including the RAS-MAPK and PI3K-AKT pathways [232]. Its function as a convergent point for diverse oncogenic signals positions SHP-2 as a high-potential therapeutic target for simultaneously suppressing multiple dysregulated proliferation pathways. SHP-2 gain-of-function alterations drive tumorigenesis, and pharmacologic inhibition of this phosphatase attenuates tumor-cell proliferation and survival [233]. Despite there being no current clinical approvals for SHP-2 inhibitors, several candidates are in clinical development. Agents Under Clinical Investigation: TNO-155 is the most advanced, currently leading the SHP-2 race in Phase II clinical trials [234]. The overall landscape of drugs targeting this specific molecular target is detailed in Table 5.

Based on the available public data summarized in Table 5, the clinical landscape for novel agents in advanced solid tumors shows few definitive positive efficacy signals, with most results still pending. A notable exception is the phase II study of DKN-01 (NCT05480306) in second-line MSS mCRC. Although the overall population did not achieve statistical significance, patients with high DKK1 expression derived substantial clinical benefit, with improved mPFS (9.0 versus 7.1 months), mOS (not reached versus 14.4 months), and ORR (38.0% versus 23.7%). These findings highlight the importance of biomarker-driven patient selection. In contrast, the clinical relevance of other ongoing trials, including those for BKM120 (NCT01591421), MEN1611 (NCT04495621), INCB050465 (NCT02646748), Nab-Rapamycin (NCT03439462), TNO155 (NCT04330664), and JAB-3068 (NCT03565003), cannot yet be assessed, as key efficacy and safety data have not been released.

### PARP inhibitors

Targeting defects in DNA repair mechanisms is a strategic complement to conventional cell proliferation inhibitors for CRC therapy. Poly (ADP-ribose) polymerase (PARP), a key orchestrator of DNA repair, is a promising target for CRC, particularly in patients with underlying DNA repair deficiencies. The following presents a systematic overview of PARP (Poly (ADP-ribose) polymerase) inhibitors, categorized by their developmental stage.

Agents under clinical investigation: PARP inhibitors employ the principle of synthetic lethality, proving most effective against tumors harboring BRCA1/BRCA2 mutations [235]. Approved drugs such as olaparib and rucaparib are currently expanding their therapeutic [236]. Nonetheless, the utility of existing inhibitors is constrained by two critical issues: (1) the common development of acquired resistance, which compromises long-term efficacy, and (2) significant dose-limiting adverse reactions such as myelotoxicity, fatigue, and gastrointestinal toxicity [237]. Next-generation PARP-targeted drugs should be designed to overcome resistance and mitigate these toxicities. Several PARP inhibitors are being evaluated in clinical trials for CRC. For instance, mechanistic studies have shown that in CRC stem cells, the PARP inhibitor veliparib (ABT-888) enhances the cytotoxic effect of 5-FU by deregulating MSH6 and subsequently inhibiting the mismatch repair (MMR) pathway [238]. Ongoing clinical trials for PARP inhibition are summarized in Table 5.

Two completed clinical trials have provided insights into the differential efficacy of olaparib in advanced CRC. The NCT04456699 study, which included a broad patient cohort without specific biomarker selection showed a survival benefit for olaparib monotherapy (mOS 21.6 months) and olaparib with bevacizumab (mOS 21.2 months) compared to standard chemotherapy (19.9 months). In contrast, the NCT04166435 trial, which targeted MGMT hypermethylation, reported a tumor ORR 0% for olaparib plus temozolomide but achieved a 55.6% DCR, demonstrating significant tumor stabilization. These findings indicate that the clinical benefit of olaparib depends primarily on the treatment regimen and patient selection: it confers modest survival gains in unselected populations but mainly achieves disease stabilization in defined molecular subgroups.

### Multitarget combination therapy

Monotherapy frequently induces drug resistance, often via the activation of alternative or parallel signaling pathways, which allows cancer cells to evade single-agent effects. For patients with advanced or mCRC, monotherapy efficacy is inadequate for achieving long-term disease control. We have also performed a comparative analysis of existing clinical drugs for CRC to clarify their respective advantages, limitations, and applicable scenarios in clinical practice. This systematic comparison encompasses major therapeutic avenues, including agents targeting the EGFR and VEGF pathways, specific mutations in BRAF V600E and KRAS G12C, HER2 amplification, NTRK fusions, ICIs for dMMR/MSI-H tumors, and broader multikinase inhibitors. The table serves as a practical reference for matching targeted treatment strategies with specific molecular subtypes and clinical contexts in CRC [239–243] (Table 6). Therefore, combining agents that target distinct pathways or multiple nodes in the same pathway is crucial to obtain synergistic antitumor effects, inhibit resistance, and ultimately improve clinical outcomes.

**Table 6:** Comparison of Current Clinical Targeted Inhibitors

Core Target/Pathway	Target/Representative Agents	Key Biomarker/Mutation Applicable Molecular Subtype (CMS)/Features	Treatment Efficacy & Key Clinical Characteristics	Primary Stage of Use
EGFR → RAS-RAF-MAPK	Anti-EGFR Agents (Cetuximab, Panitumumab)	RAS (KRAS/NRAS) wild-type; BRAF V600E wild-type CMS2 (Canonical) most sensitive. Left-sided tumors show better outcomes	Improves OS/PFS in RAS wild-type mCRC when combined with Chemotherapy Use restricted to left-sided, RAS/BRAF wild-type tumors	1st line & later lines [244–246]
VEGF/VEGFR → Angiogenesis	Anti-VEGF/VEGFR Agents (Bevacizumab, Aflibercept, Ramucirumab)	No specific mutation required (Broad-spectrum) Applicable to all subtypes	Foundation of 1st-line therapy, extending survival. Efficacy independent of tumor location. Monitor HTN, proteinuria	1 st line, maintenance & later lines [247–250]
MAPK Pathway (BRAF V600E)	BRAF V600E Inhibitors (Encorafenib + Cetuximab)	BRAF V600E mutation (~ 8–10% of mCRC) Often CMS1 or CMS3, but most are MSS. More common in right-sided colon	BRAF inhibitor monotherapy ineffective. Must combine with an EGFR inhibitor	Later lines [241, 251]
MAPK Pathway (KRAS G12C protein)	KRAS G12C Inhibitors (Sotorasib, Adagrasib)	KRAS p.G12C mutation (~ 3–4% of mCRC) Not limited by location	Limited efficacy as monotherapy (ORR ~ 10%). Combination with EGFR inhibitor improves efficacy (ORR ~ 30%)	Later lines [252]
NTRK gene fusion → TRK protein	NTRK Inhibitors (Larotrectinib, Entrectinib)	NTRK1/2/3 gene fusion (< 1% in CRC) Not restricted	Highly effective for fusion-positive tumors. Requires screening via NGS/FISH	Later lines [253, 254]
HER2 → MAPK/PI3K	HER2-Targeted Agents (Trastuzumab + Pertuzumab, T-DXd)	HER2 amplification/overexpression (~ 2–5% of RAS/BRAF WT mCRC) Not restricted	For HER2-positive mCRC after standard therapy. High response rates with dual blockade/ADCs	Later lines [255]
PD-1/PD-L1/CTLA-4	ICIs (Pembrolizumab, Nivolumab ± Ipilimumab)	dMMR/MSI-H Predominantly CMS1. Associated with right-sided colon cancer	Revolutionary for MSI-H/dMMR mCRC. Largely ineffective in MSS/pMMR tumors	1st line, neoadjuvant for MSI-H/dMMR mCRC [256]
VEGFR1-3, PDGFR, FGFR, RAF, etc	Multi-Kinase Inhibitors (Regorafenib, Fruquintinib)	No specific mutation required All subtypes, especially MSS-type in later lines	Clear survival benefit. Manage side effects (hand-foot reaction, HTN, fatigue)	3rd line, beyond after standard therapy [257]
Thymidylate Synthase Inhibitor	Cytotoxic Agent (TAS-102)	No specific mutation required All subtypes	Superior survival vs. placebo in later-line. Myelosuppression is main side effect	3rd line &, beyond after standard therapy [258]

The rapid adoption of combination therapy strategies offers renewed hope for patients with advanced cancer. A study assessing garsorasib combined with cetuximab in KRAS G12C- mutated advanced CRC demonstrated significantly improved ORR and PFS with a favorable safety profile, paving the way for phase III trials [259]. In immunotherapy, the combination of nivolumab plus ipilimumab significantly prolonged PFS compared with monotherapy in MSI-H or MMR-deficient patients, irrespective of prior therapy, suggesting that it could emerge as a new standard of care considering its manageable safety [260]. A study investigating an intermittent dosing strategy of FOLFIRI plus panitumumab following induction therapy in unresectable RAS/BRAF wild-type mCRC demonstrated a significantly higher 12-month PFS on treatment (PFSot) rate in the intermittent group (Arm B) compared with the continuous treatment arm (Arm A) (58.5% versus 45.7%). The intermittent approach extended median PFS to 17.5 months and reduced the incidence of severe skin adverse events, showing promise for improving quality of life. Independently, for BRAF V600E- mutant mCRC, the combination of encorafenib plus cetuximab with mFOLFOX6 in previously untreated patients significantly improved ORR (60.9% versus 40.0%) and median duration of response (13.9 versus 11.1 months), with a safety profile consistent with individual agents, establishing it as a highly promising therapeutic option [261]. The integration of multimodal strategies, such as targeted therapy, immunotherapy, and microenvironment/gut microbiota modulation, has significantly improved CRC treatment efficacy and prognosis. Achieving optimal results requires precision combination therapy, dependent on advanced technologies for predicting patient response and improving regimens. Successful treatment is contingent on identifying the appropriate patient subpopulation, as shown by the efficacy of dual HER2 blockade in HER2-amplified mCRC [262, 263]. Patient-derived organoids are emerging as essential patient-specific ex vivo models capable of predicting individual patient responses and informing personalized combination strategies [264]. Clinical trials evaluating the outcomes of these promising combination regimens in CRC are detailed in Table 7.

**Table 7:** Multitarget combination therapy under clinical investigation of CRC

Name or ID	CRC subtype	Phase	PFS	NCT identifier
MRG003	Wild type	I	NA	NCT04868344 [265]
Garsorasib/Cetuximab	KRAS G12C mutant	II	7mouth	NCT04585035 [259]
Durvalumab/Tremelimumab/mFOLFOX6	MSS metastatic RAS mutant unresectable metastatic	1b/II	8.53 months	NCT03202758 [266]
Encorafenib/Binimetinib/Cetuximab	BRAFV600E mutant metastatic	II	5.8 months	NCT03693170 [267]
SBRT/Atezolizumab	Wild type	II	1.4 months	NCT02992912 [268]
Sorafenib/SBRT	Metastatic	II	NA	ChiCTR2200066117 [269]
Cabozantinib/ICI durvalumabin	pMMR/MSS advanced	II	6.3 months	NCT03539822 [270]
Bevacizumab/Ramucirumab	RAS mutant	II	9.2 months	NCT01079780 [271]
Cabozantinib/Durvalumab	pMMR/MSS	I/II	4.5 months	NCT03539822 [272]
Regorafenib/Nivolumab	pMMR	I/Ib	4.3 months	NCT03712943 [273]
CXD101/Nivolumab	MSS	II	2.1 months	NCT01092481 [274]
AZD8931/irinotecan/5-FU (FOLFIRI)	mCRC	I/II	8.7 months	NCT01092481 [275]
Pertuzumab/Trastuzumab	HER2 positive	II	17.2 weeks	NCT02693535 [276]
Divarasib/Cetuximab	KRAS G12C positive	Ib	8.1 mouths	NCT04449874 [277]
Encorafenib/Cetuximab/Binimetinib	BRAFV600E mutant metastatic	III	5.3 mouths	NCT02928224 [278]
Sparatlizumab/Dabrafenib/Trametinib	BRAFV600E	II	4.3 months	NCT03668431 [279]
Serplulimab/HLX04/Capecitabine/Oxaliplatin	MSS	II/III	17.2 months	NCT04547166 [280]
Pembrolizumab/XL888	Advanced	Ib/II	1.9 months	NCT03095781 [281]
Nivolumab/Ipilimumab	MSI metastatic	III	54.1 months	NCT04008030 [260]
FOLFIRI/Cetuximab/Bevacizumab	RAS wild type metastatic	III	12.6 months	NCT00433927 [282]
Nivolumab/Ipilimumab	MSI/dMMR metastatic	II	3 year 72.0% 5 year 65.3%,	NCT03350126 [283]
Regorafenib/Sintilimab/Encorafenib	MSS metastatic	II	4.1 months	NCT04745130 [53]
Cetuximab/mFOLFOX6	BRAF mutant	III	13.9 months	NCT04607421 [261]
Sotorasib/Panitumumab	KRAS G12C mutant metastatic	III	5.7 months	NCT05198934 [284]
Etuximab β/FOLFIRI	RAS/BRAF wild type metastatic	III	13.1 months	NCT03206151 [285]
Panitumumab/Fluorouracil/Leucovorin/Irinotecan	RAS/BRAF wild type metastatic	II	11.2 months	NCT04425239 [286]

With ongoing research, an increasing number of drugs and treatment approaches have been developed. However, due to the inherent complexity of both the human body and tumors themselves, as well as the notable diversity and heterogeneity among them, patients vary significantly in their response to the same drug, which continues to present a series of serious challenges in the treatment of CRC.

## Current challenges and future directions

Although molecular research in CRC has driven significant advances in targeted therapy, major clinical challenges persist [287]. These challenges include the development of acquired treatment resistance and pronounced variability in patient responses, which complicate the identification of appropriate candidates. The core issue is the need for more specific predictive biomarkers to ensure efficacy [173]. Additionally, the high financial burden of these specialized agents and the complexity of combination regimens restrict patient accessibility and adherence, underscoring the necessity for sustained efforts to optimize targeted strategies and enhance patient outcomes.

### Tumor heterogeneity and clonal evolution

Tumor heterogeneity is a major barrier to the efficacy of targeted and combination therapies, driving drug resistance and treatment failure in CRC [288]. This highly heterogeneous and complex disease exhibits variability across tumor types, stages, locations, and at the core molecular level [289]. The heterogeneity can be systematically defined across four dimensions. 1) Genetic heterogeneity: during clonal expansion, CRC cells accumulate a vast number of somatic mutations, resulting in distinct genetic landscapes both within (intra-tumoral) and between (inter-patient) tumors. Colantuoni’s research team revealed a CRC somatic mutation burden several times higher than that of normal tissue. This heterogeneity is observed not only across different patients but also within distinct regions of a single tumor and it evolves across different stages, posing a fundamental challenge to unified targeted therapy [290]. 2) Epigenetic heterogeneity: a key finding from Bao et al. was the generally lower genome-wide DNA methylation observed in CRC tumors relative to neighboring normal epithelium [291]. Remarkably, distinct subclones within the same tumor tissue also exhibit differential methylation levels. Furthermore, post-translational modifications such as protein acetylation play a major role in CRC metastasis, as evidenced by substantial differences in acetylated protein expression levels between primary CRC and liver metastatic lesions [292]. 3) Transcriptomic heterogeneity: a high-resolution analysis using single-cell RNA sequencing systematically depicted the dynamic changes and molecular profiles of up to 48 distinct cell subtypes during CRC progression [293]. The subtypes exhibited distinct gene expression patterns, highlighting significant inter-group differences that were closely correlated with fundamental tumor biological behaviors, including cell proliferation, invasion, and intrinsic sensitivity to treatment [294]. 4) Functional/phenotypic heterogeneity: divergence in fundamental biological traits, including proliferation capacity, the utilization of specific metabolic pathways, and overall metastatic potential, collectively dictates the trajectory of tumor progression and the ultimate outcome of chemotherapy [295].

The complex nature of CRC heterogeneity can be classified as intratumoral and intertumoral heterogeneity. Intratumoral heterogeneity refers to differences among cancer cells within a single tumor, including spatial heterogeneity (variations across physical regions) and clonal heterogeneity (the coexistence of multiple subclones, each with distinct genetic characteristics) [296]. In contrast, intertumoral heterogeneity highlights significant differences between the same tumor type in different patients, such as varying driver gene mutations, growth kinetics, and drug sensitivities, emphasizing the challenge and importance of precision medicine [297]. This multilevel heterogeneity, both within and between patients, necessitates personalized and dynamic treatment regimens [298]. Considering the complex, evolving nature of CRC, single-target therapies fail frequently [299]. Therefore, future strategies must incorporate multitargeted therapies along with continuous genetic monitoring to achieve durable clinical responses [300, 301].

### Mechanisms of drug resistance

Drug resistance, the central driver of CRC treatment failure, is categorized into primary and acquired forms [302]. 1) Primary resistance- the lack of initial response- is mechanically complex, often driven by genetic mutations [303]. For example, RAS mutations confer resistance to cetuximab by aberrantly and continuously promoting proliferation and survival signals, thereby overcoming the inhibitory effect of this drug on tumor growth [304], Quantitatively, RAS mutations (KRAS/NRAS) are present in approximately 50% of metastatic CRC cases and are a predominant mechanism of primary resistance to EGFR inhibitors, with over 90% of patients harboring these mutations deriving no clinical benefit from such therapies [305]. Specific subtypes like the KRAS G12C mutation, found in about 3% of mCRC, confer distinct resistance profiles [306]. Phenotypic changes, such as the acquisition of stem cell-like properties (e.g., enhanced self-renewal), further contribute to this intrinsic drug tolerance [307]. 2) Acquired resistance develops when tumors gradually become resistant after a period of effective treatment. The genetic and phenotypic heterogeneity of tumors jointly promotes the development of acquired resistance [308]. Tumor cells evolve genetically, with some harboring pre-existing resistance mutations that confer an immediate adaptive advantage and allow rapid dominance within the tumor population [309]. Acquired resistance manifests as tumor relapse following a period of effective treatment, propelled by genetic and phenotypic evolution [308]. Longitudinal genomic studies reveal that in patients initially responding to anti-EGFR therapy, acquired resistance is driven by the emergence of detectable RAS mutations or EGFR extracellular domain (ECD) mutations in approximately 30% to 70% of cases [310]. Therapeutic pressure selects resistant clonal cells via two paths: the rapid dominance of clones with pre-existing resistance mutations or, in their absence, reliance on dynamic phenotypic adaptations [311]. These adaptations, including transcriptional priming and epigenetic plasticity, initiate upon drug exposure, leading to the formation of pre-drug-tolerant persistent cells (pre-DTP) [312]. These cells eventually transition from non-proliferating to proliferating DTP cells, ensuring tumor cell survival and progression under continuous therapy. Quantitative models suggest that DTP populations can constitute a small but significant fraction (e.g., ~ 0.3%−5%) of the tumor cell population at the onset of treatment, serving as a reservoir for eventual relapse [313].

Drug resistance is a critical, multimechanistic challenge in cancer treatment, involving both endogenous and exogenous factors [314]. The development of acquired resistance to anti-EGFR monoclonal antibodies often involves the acquisition of secondary mutations in downstream signaling components, including KRAS, NRAS, and BRAF, which prevent the drug from effectively binding to its target and maintaining constitutive signaling [304]. Similarly, prolonged use of BRAF inhibitors in BRAF V600E-mutant tumors can lead to resistance through the compensatory activation of alternative signaling pathways [315, 316]. Resistance to anti-HER2 therapies is also linked to the abnormal activation of parallel signaling branches, such as PI3K-AKT activation resulting from HER2 amplification, which bypasses the intended inhibitory signal [317]. Autophagy plays a context-dependent role in tumorigenesis, capable of functioning as either a tumor suppressor or a tumor promoter. This functional switch is modulated by genetic and environmental factors through key signaling pathways, such as the PI3K-AKT-mTOR axis and AMPK [318]. Mechanistically, TIPE3 has been shown to enhance autophagy by upregulating USP19 expression, affecting Beclin1 protein levels, and thereby inducing resistance to targeted drugs [319].

Extrinsic factors from the TME also play a significant role in drug resistance [320]. The accumulation of lactate creates an acidic, nutrient- and oxygen-deficient metabolic environment that promotes tumor progression, metastasis, and triggers specific resistance mechanisms [321]. For instance, histone H3K18la, an epigenetic modification, activates rubicon-like autophagy enhancer to enhance autophagy, leading to resistance against bevacizumab [322]. Additionally, the cholesterol metabolite 27-hydroxycholesterol (27HC) mediates resistance through diverse pathways, including promotion of drug efflux, cell proliferation, inhibition of apoptosis, induction of EMT, and metabolic reprogramming [323]. CRC resistance is thus a complex consequence of integrated factors and pathways. Effective resistance-reversing strategies must comprehensively account for these TME-driven and metabolic mechanisms to achieve precision and efficacy.

### Tumor microenvironment and immune evasion

TME, shaped by microbial factors and host immune interactions, critically drives carcinogenesis via immune evasion. This process generates genomic instability, promotes aberrant signaling, and fosters an immunosuppressive ecosystem by recruiting inhibitory cells [46]. Tumor cells evade immune surveillance through several key mechanisms: (1) downregulating antigen presentation (e.g., loss of MHC expression) to prevent T-cell recognition; (2) actively shaping the TME by secreting factors and recruiting immunosuppressive cells, such as Tregs; and (3) upregulating immune checkpoint molecules (e.g., PD-L1), leading to T-cell exhaustion. Collectively, these strategies weaken the antitumor immune response and promote tumor growth [324]. These mechanistic insights have successfully underpinned the development of immunotherapies, such as ICIs, that reverse immune escape by blocking inhibitory signals, opening new therapeutic avenues.

ICIs are revolutionizing CRC treatment through their mechanism of blocking immune evasion pathways. This class includes agents targeting CTLA-4 (e.g., ipilimumab), PD-1 (e.g., pembrolizumab and nivolumab), and PD-L1 (e.g., atezolizumab) [325]. The pivotal KEYNOTE-177 and CheckMate-142 studies have established ICIs as the cornerstone therapy for metastatic CRC with MSI-H/dMMR status. Specifically, the Phase III KEYNOTE-177 trial showed that first-line pembrolizumab significantly improved PFS alongside a more favorable safety profile than standard chemotherapy [326]. The Phase II CheckMate-142 study confirmed that Nivolumab plus Ipilimumab achieved durable and high ORRs in previously treated patients, offering an effective, chemotherapy-free approach for subsequent lines of therapy [260].

Despite breakthroughs in immunotherapy for MSI-H/dMMR tumors, significant therapeutic challenges persist for the majority of CRC patients (~ 95%) with microsatellite-stable/mismatch repair proficient (MSS/pMMR) status. These tumors typically feature a noninflammatory or “immune-excluded” TME, characterized by a low TMB, insufficient T-cell infiltration, and the active role of immunosuppressive cells [327, 328]. Clinical data, including the CheckMate-142 trial, have confirmed that neither monotherapy nor standard dual immune checkpoint inhibitor therapy induces meaningful responses in this MSS population [329]. The TME in these tumors is often dominated by immunosuppressive interactions, such as those involving T-bet⁺ Treg cells, which suppress CD8⁺ T-cell function via high CD39 expression [330]. Targeting these key regulatory cells represents a promising strategy to enhance antitumor immunity and improve the efficacy of immune therapies.

To overcome the resistance of MSS CRC to immunotherapy, rational combination strategies aimed at converting the “cold” noninflammatory TME, into an immune-infiltrated, “hot” microenvironment are being developed. The key approaches involve combining ICIs with agents that actively modulate the immunosuppressive landscape. 1) Multikinase inhibitors: agents like Regorafenib exhibit preclinical efficacy by disrupting pro-tumorigenic factors such as TAMs and angiogenic pathways. This modulation provides a solid rationale for combination with ICIs to boost antitumor immunity. 2) Novel immunomodulatory targets: the peptidyl prolyl isomerase Pin1, highly expressed in MSS CRC, drives immunosuppression through the NF-κB-CCL3-CCR5 axis. Preclinical studies indicate that Pin1, which is inhibition enhances anti-PD-1 efficacy, representing a promising new therapeutic target. 3) Investigational strategies also include combining ICI with conventional treatments (chemotherapy, radiotherapy) or novel targeted agents to achieve synergistic immune priming and checkpoint blockade sensitization. A study has developed a bispecific antibody, ATAPL1, that simultaneously targets PD-L1 and TNFR2. It demonstrated excellent tumor-targeted accumulation and antitumor efficacy in a mouse model of CRC, with no significant toxicity. Its mechanism of action involves remodeling the tumor immune microenvironment, reducing immunosuppressive cells, activating CD8⁺ T cells and macrophages, and initiating long-term immune surveillance, providing a promising new strategy for CRC treatment [331]. Research on tumor vaccine development is also progressing, such as targeting tumor-associated antigens (e.g., CEA) or shared neoantigens (e.g., KRAS mutations); designing mRNA or peptide vaccines based on patient tumor mutation profiles; or inducing systemic anti-tumor immunity through intratumoral injection of immunostimulants (e.g., Flt3L, radiotherapy). CRC vaccines still face challenges such as tumor heterogeneity, antigen escape, and the immunosuppressive microenvironment, but strategies combining them with ICIs show potential [332]. Another study developed a recombinant oncolytic influenza virus, rPR8-CCL19, carrying the chemokine CCL19 for the treatment of CRC. This virus selectively infects and kills CRC cells, expresses CCL19 in the tumor microenvironment, recruits and activates dendritic cells and T cells, converts “cold” tumors into “hot” tumors, effectively inhibits tumor growth and metastasis, and induces systemic tumor-directed immunity and persistent immunologic memory, with a good safety profile [333].

### Identifying and validating novel biomarkers

Current mCRC management relies on established biomarkers (RAS mutations, BRAF V600E, MSI status) to guide the use of anti-EGFR agents, BRAF/EGFR combinations, and ICIs [334–336]. However, this static approach fails to capture crucial temporal heterogeneity and clonal evolution, driving acquired resistance [337]. Moreover, it overlooks critical disease dimensions, including transcriptomics, proteomics, and the TME, limiting efficacy [338]. These constraints underscore the urgent need for dynamic, multidimensional biomarkers to optimize treatment selection and advance CRC precision oncology.

The integration of advanced technologies is driving a new era of dynamic CRC biomarker discovery. 1) Liquid biopsy: Circulating tumor DNA (ctDNA) allows for noninvasive, real-time monitoring, with its most impactful application being the detection of minimal residual disease post-surgery. The DYNAMIC trial has shown that ctDNA-guided management can safely reduce the adjuvant chemotherapy burden in stage II colon cancer [339]. ctDNA is also vital for early detection of acquired resistance mutations [340]. 2) Multi-omics and artificial intelligence: multiple multi-omics approaches, such as the transcriptomic CMS classification, identify biologically relevant subgroups (e.g., stromal-rich CMS4 tumors) [341, 342]. Moreover, proteogenomics can also reveal dysregulated mechanisms missed by genomics [343, 344]. Concurrently, AI applied to digital pathology offers a cost-effective tool to predict molecular characteristics (e.g., MSI, BRAF status) [345]. 3) Tumor Microbiome: intratumoral microbes (e.g., *Fusobacterium nucleatum*) have emerged as prognostic factors correlated with poor survival and chemoresistance [346, 347]. The translation of these novel biomarkers into routine practice faces multiple challenges. Tumor heterogeneity limits the representativeness of a single sample, and technical standardization is required for assay reproducibility (e.g., ctDNA detection, microbiome analysis). The clinical utility of these novel biomarkers must be established via prospective, randomized trials before clinical adoption.

The future of CRC biomarker development and therapy is exceptionally promising. Key directions involve aggressively targeting historically challenging drivers. Following the success of KRAS G12C inhibitors, efforts are focused on other mutants, exemplified by MRTX1133 (KRAS G12D) entering clinical trials [348]. Moreover, technologies like Proteolysis-targeting chimeras offer a versatile strategy to degrade, rather than inhibit, problematic oncoproteins, potentially accessing previously “undruggable” drivers such as transcription factors [349]. Additionally, for the resistant MSS/pMMR CRC majority, neoantigen-based vaccines and engineered cell therapies are in development to induce de novo immune responses, with initial trials demonstrating strong immunogenicity [349, 350].

### Rational design of combination therapies

Overcoming the limitations of targeted monotherapies in CRC requires a detailed mechanistic understanding of tumor biology and adaptive resistance. The inherent tumor heterogeneity and high rate of therapeutic failure necessitate a shift toward rational combination design aimed at creating synergistic and durable outcomes [351]. This combination employs vertical and horizontal inhibition logic to block tumor escape mechanisms, vertical inhibition involves parallel blockade of a single, central pathway. The treatment of BRAF V600E-mutant mCRC is a paradigm: monotherapy resistance is driven by rapid EGFR feedback activation. The rationally engineered triple combination (BRAF + EGFR + MEK inhibitors) achieves a potent vertical MAPK pathway blockade with remarkable clinical benefit [315, 351, 352]. Moreover, horizontal inhibition combines targeted therapy with distinct mechanisms like immunotherapy. The multi-kinase inhibitor Regorafenib exemplifies this by modulating the immunosuppressive TME. Its combination with Nivolumab (PD-1 inhibitor) showed initial activity in MSS mCRC, a cohort typically resistant to immunotherapy [353]. The discovery of novel synergistic drug pairs and predictive biomarkers is being significantly accelerated by AI-powered large-scale drug screening platforms [354].

Significant challenges limit the impact of current combination strategies, notably managing cumulative toxicity and preventing adaptive resistance. Future success hinges on rigorous, dynamic resistance monitoring and the clinical adoption of efficient, innovative approaches. Adaptive clinical trial designs (e.g., platform trials) are crucial for evaluating multiple combinations concurrently within a standardized framework [355, 356]. Ultimately, integrating deep molecular insights with advanced functional and computational technologies is the necessary path for developing truly personalized, rationally designed combination regimens for CRC.

## Conclusion and outlook

This review systematically summarizes the advancements in molecular pathogenesis, targeted therapy, and immunotherapy of CRC, analyzing the core limitations of current management. Despite its high incidence and mortality, CRC care has been revolutionized by the development of targeted therapies. We identified key signaling pathways, including Wnt/β-catenin, PI3K/AKT/mTOR, EGFR, JAK/STAT, VEGF, and AMPK, as crucial drivers and therapeutic targets. The category of targeted agents encompasses key drugs such as cetuximab (an anti-EGFR monoclonal antibody), encorafenib (a BRAF inhibitor), and bevacizumab (a VEGF inhibitor), along with ICIs for MSI-H disease, have fundamentally transformed mCRC outcomes. Nevertheless, significant therapeutic hurdles remain, including, among others, tumor heterogeneity and the emergence of both primary and acquired drug resistance. These obstacles, compounded by economic constraints, necessitate a sustained research focus on elucidating resistance mechanisms, validating next-generation predictive biomarkers, and developing innovative, resistance-reversing therapeutic combinations.

Despite significant progress in understanding the molecular basis of CRC, interconnected obstacles persist, limiting the clinical impact of precision oncology. These challenges mandate continued, focused research into resistance mechanisms, predictive biomarkers, and innovative therapeutic approaches. The intrinsic heterogeneity of CRC, spanning both inter-tumor differences and intra-tumor clonal diversity, fundamentally complicates the development of universal therapeutic strategies. Technologies like spatial multiomics are key to resolving this heterogeneity, but require further maturation to overcome current limitations in resolution and data integration. Progress in bioinformatics and computational biology is thus paramount for developing sophisticated analytical tools necessary to derive clinical meaning from vast multi-omics data. Furthermore, achieving global equity in precision oncology is restricted by the significant economic burden. The high costs of high-throughput multi-omics analyses and subsequent personalized drugs pose major obstacles. This is compounded by the lack of human-relevant preclinical models that faithfully represent the CRC TME, underlining the need for advanced model systems to bridge the bench-to-bedside translational gap.

Addressing the global health challenge posed by CRC requires interdisciplinary collaboration, uniting wet-lab scientists, clinicians, and bioinformaticians. Sustained commitment to research and development is indispensable for the successful assimilation of advanced technologies into clinical practice. The future of CRC is intricate, requiring a dynamic, personalized, and patient-centered strategy. The integration of AI technologies into oncology will introduce new possibilities for precision medicine in diagnosis and treatment planning. By combining targeted therapy with AI, informed by deep molecular knowledge of CRC pathogenesis, we can establish the foundation for significantly improved treatment regimens. Through such focused, collaborative endeavors, it is plausible to realize the potential of precision oncology, advancing the field and offering hope worldwide by making CRC a manageable condition.

The integration of targeted therapy with immunotherapy, microenvironment modulation, and microbial intervention constitutes a powerful multimodal therapeutic strategy. Enhanced precision in patient care is expected through molecular subtyping and personalized medicine, facilitating better identification of those most likely to benefit. However, fundamental challenges persist, including the emergence of drug resistance and the limited effectiveness of single-agent therapies. While combination approaches offer theoretical benefits, complexities like unpredictable drug interactions and high interpatient variability often limit clinical efficacy and can exacerbate toxicity. Deeper mechanistic studies are required to fully elucidate the synergistic mechanisms underlying these combinations, thereby optimizing therapeutic effects while mitigating adverse reactions.

## Data Availability

The authors have nothing to report.

## Abbreviations

ADC: Antibody-Drug Conjugate
ADCC: Antibody-Dependent Cell-Mediated Cytotoxicity
ADCs: Antibody-drug conjugates
AMPK: Adenosine Monophosphate-Activated Protein Kinase
APC: Adenomatous polyposis coli
ATP: Adenosine triphosphate
BCL2: B-cell Lymphoma 2
BHB: β-Hydroxybutyrate
CAC: Colitis-Associated Colorectal Cancer
CIMP: CpG island methylator phenotype
CIMP-H: CIMP-High
CIN: Chromosomal instability
c-Met: Mesenchymal-epithelial transition factor
CMS: Consensus Molecular Subtypes
CRC: Colorectal cancer
CSC: Cancer Stem Cell
ctDNA: Circulating tumor DNA
DHODH: Dihydroorotate dehydrogenase
dMMR: Mismatch repair deficiency
DTP: Drug-tolerant persistent cells
Dvl: Dishevelled
ECD: Extracellular domain
ECM: Extracellular matrix
EGFR: Epidermal growth factor receptor
EIF3H: Eukaryotic initiation factor 3 subunit H
EMT: Epithelial-mesenchymal transition
FOSL1: Fos-Like Antigen 1
4-HPA: 4-Hydroxyphenylacetic acid
5-FU: 5-Fluorouracil
FDA: Food and Drug Administration
FGF1: Fibroblast Growth Factor 1
FGFR: Fibroblast growth factor receptor
FUT4: Fucosyltransferase 4 / Fucosyl Transferase 4
FZD: Frizzled
GLTSCR1: Glioma Tumor Suppressor Candidate Region Gene 1
HCC: Hepatocellular carcinoma
HIF-1α: Hypoxia-inducible factor 1-alph
IARC: International Agency for Research on Cancer
IBD: Inflammatory bowel diseas
IFN-γ: Interferon-γ
LMR: Lymphocyte-to-monocyte ratio
mCRC: Metastatic CRC
MDSCs: Myeloid-derived suppressor cells
miR-26a/26b: MicroRNA-26a/26b
MMR: Mismatch Repair
MSI: Microsatellite instabilit
MSS: Microsatellite-stable
NECD: Notch extracellular domai
NFAT: Nuclear factor of activated T cells
NF-κB: Nuclear factor kappa B
NICD: Notch intracellular domain
NSCLC: Non-small cell lung cancer
ORR: Objective response rate
OS: Overall survival
PARP: Poly ADP-Ribose Polymerase
PFS: Progression-free survival
PFSot: Progression-Free Survival on Treatment
PLGF: Placental Growth Factor
PRELP: Proline/Arginine-Rich End Leucine-Rich Repeat Protein
RTKs: Receptor Tyrosine Kinases
RUBCNL: Rubicon Like Autophagy Enhancer
SHP-2: Src-Homology-2 Domain-Containing Phosphatase 2
SLITRK4: SLIT and NTRK-like Family Member 4
TAMs: Tumor-Associated Macrophages
T-DXd: Trastuzumab deruxtecan
TI-HTN: Treatment-induced hypertension
TIMP-2: Tissue inhibitor of Metalloproteinases-2
TKI: Tyrosine Kinase Inhibitor
TMD: Transmembrane domain
TME: Tumor microenvironment
TNF: Tumor necrosis factor
Tregs: Regulatory T cells
VEGF: Vascular endothelial growth factor / Vascular Endothelial Growth Factor
WRN: Werner syndrome RecQ helicase
ZNF217: Zinc-finger protein 217
ORR: Objective Response Rate
CR: Complete Response
PR: Partial Response
DCR: Disease Control Rate
CBR: Clinical Benefit Rate
BOR: Best Overall Response
DOR: Duration of Response
TTR: Time To Response
PFS: Progression-Free Survival
DFS: Disease-Free Survival
AE: Adverse Even
TEAE: Treatment-Emergent Adverse Event
TRAE: Treatment-Related Adverse Event
SAE: Serious Adverse Event
DLT: Dose Limiting Toxicity
AESI: Adverse Event of Special Interest
MTD: Maximum Tolerated Dose
RP2D: Recommended Phase 2 Dose
MAD: Maximum Administered Dose
OBD: Optimal Biological Dose
RD: Recommended Dose
TD: Target Dose
Cmax: Maximum (Plasma) Concentration
Tmax: Time to Cmax
TSR: Treatment Success Rate
RAD: RECIST-Assessed Disease
BRR: Best Response Rate
